# A UHPLC-Mass Spectrometry View of Human Melanocytic Cells Uncovers Potential Lipid Biomarkers of Melanoma

**DOI:** 10.3390/ijms222112061

**Published:** 2021-11-08

**Authors:** Arantza Perez-Valle, Beatriz Abad-García, Olatz Fresnedo, Gabriel Barreda-Gómez, Patricia Aspichueta, Aintzane Asumendi, Egoitz Astigarraga, José A. Fernández, María Dolores Boyano, Begoña Ochoa

**Affiliations:** 1 Department of Cell Biology and Histology, Faculty of Medicine and Nursing, University of the Basque Country UPV/EHU, 48940 Leioa, Spain; arantza.perezv@ehu.eus (A.P.-V.); aintzane.asumendi@ehu.eus (A.A.); 2 Central Analysis Service, Faculty of Science and Technology, University of the Basque Country UPV/EHU, 48940 Leioa, Spain; beatriz.abad@ehu.eus; 3 Department of Physiology, Faculty of Medicine and Nursing, University of the Basque Country UPV/EHU, 48940 Leioa, Spain; olatz.fresnedo@ehu.eus (O.F.); patricia.aspichueta@ehu.eus (P.A.); 4 IMG Pharma Biotech S.L., Bizkaia Technological Park, 48160 Derio, Spain; gabriel.barreda@imgpharma.com (G.B.-G.); egoitz.astigarraga@imgpharma.com (E.A.); 5 Biocruces-Bizkaia Research Institute, Cruces University Hospital, 48903 Barakaldo, Spain; 6 Department of Physical Chemistry, Faculty of Science and Technology, University of the Basque Country UPV/EHU, 48940 Leioa, Spain; josea.fernandez@ehu.esu

**Keywords:** lipid biomarker, lipid phenotype, human, melanoma, melanocyte, nevus melanocyte, sphingomyelin, plasmalogen, ether lipid, metastasis

## Abstract

Melanoma is the deadliest form of skin cancer due to its ability to colonize distant sites and initiate metastasis. Although these processes largely depend on the lipid-based cell membrane scaffold, our understanding of the melanoma lipid phenotype lags behind most other aspects of this tumor cell. Here, we examined a panel of normal human epidermal and nevus melanocytes and primary and metastatic melanoma cell lines to determine whether distinctive cell-intrinsic lipidomes can discern non-neoplastic from neoplastic melanocytes and define their metastatic potential. Lipidome profiles were obtained by UHPLC-ESI mass-spectrometry, and differences in the signatures were analyzed by multivariate statistical analyses. Significant and highly specific changes in more than 30 lipid species were annotated in the initiation of melanoma, whereas less numerous changes were associated with melanoma progression and the non-malignant transformation of nevus melanocytes. Notably, the “malignancy lipid signature” features marked drops in pivotal membrane lipids, like sphingomyelins, and aberrant elevation of ether-type lipids and phosphatidylglycerol and phosphatidylinositol variants, suggesting a previously undefined remodeling of sphingolipid and glycerophospholipid metabolism. Besides broadening the molecular definition of this neoplasm, the different lipid profiles identified may help improve the clinical diagnosis/prognosis and facilitate therapeutic interventions for cutaneous melanoma.

## 1. Introduction

Melanoma is regarded as the deadliest form of skin cancer, arising after the malignant transformation of neural crest-derived melanocytes in the epidermis of the skin [1]. The transformation of these cells is due to a plethora of molecular alterations (reviewed in [2]), including activating mutations in the proto-oncogene kinase BRAF. In conjunction, these not only account for the aggressiveness of melanoma and its ability to metastasize to other organs but also the strong intratumoral heterogeneity and lack of specific biomarkers with high diagnostic accuracy. Thus, discovering new biomarkers and elucidating the molecular events underlying melanoma initiation and progression will be necessary to develop more selective and powerful therapies for melanoma patients. 

Metabolic changes are observed in most cancer cells, including accelerated nutrient import and metabolic rewiring to enhanced aerobic glycolysis [3,4,5,6,7,8,9,10]. Alterations in lipid metabolism and the development of a lipogenic phenotype have also emerged as early biochemical hallmarks of cancer cells [11,12,13,14,15,16,17,18]. In this regard, studies assessing the metabolic behavior of melanoma have demonstrated that such phenotypic plasticity confers adaptive advantages favoring proliferation and survival [4,19,20]. For instance, while the de novo biosynthesis of fatty acids (FA) is mild in normal adult tissues, the tumorigenesis-associated increase in lipid production enables cells to create a strategically structured new biomass [12,13], helping them better handle changing environmental conditions [3]. Growth and lipid metabolism are reciprocally linked through transcriptional programs operated by sterol regulatory element-binding protein-1 (SREBP-1) [21,22]. In a recent study of melanoma cells, not only was it confirmed that de novo lipogenesis relies essentially on the proteolytic activation of SREBP-1, but also, it was demonstrated that lipogenesis is a key mediator of the oncogenic tumor effects of BRAF implicated in resistance to therapy [23]. They demonstrated that while de novo lipogenesis is inhibited by BRAFv600E-targeted therapy in BRAF-mutant cells sensitive to therapy, cells resistant to therapy invariably rescue SREBP-1 processing and lipogenesis [23]. Since de novo lipogenesis is mainly responsible for the formation of saturated FA (i.e., C16:0), which are rapidly incorporated into neutral lipids and phospholipids, therapy-resistant cells exhibit an increase in membrane saturation [23]. Other features of lipid biochemistry also seem to be critical to the aggressiveness of melanoma cells, such as the enhanced provision of endogenous FA and protumorigenic signals through lipolytic monoacylglycerol lipase [14,15] and the acquisition of lipids from the extracellular space by metastasis-initiating cells through the FA receptor CD36 [24] or those from stromal adipocytes through FATP lipid transporter proteins [25]. Thus, differential lipid signatures might yield molecular biomarkers for the diagnosis and/or prognosis of melanoma, although data on the lipidome of melanoma cells are still somewhat limited.

**Figure 1:**
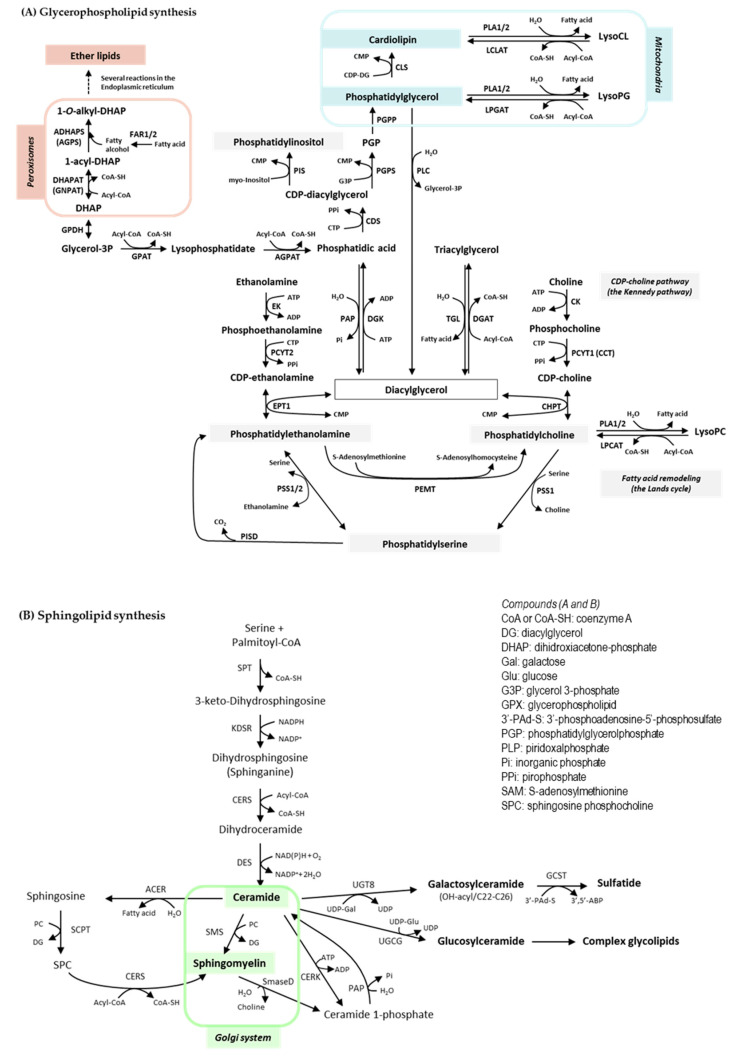
Core glycerophospholipid (**A**) and sphingolipid (**B**) biosynthetic pathways. Enzymes (**A**): ADHAPS: alkylDHAP synthase; AGPAT: acyl:glycerol-3-phosphate acyltransferase; CDS: CDPDG synthase; CHPT: CDP:phosphocholine phosphotransferase; CK: choline kinase; CLS: cardiolipin synthase; DGAT: DG acyltransferase; DGK: DG kinase. DHAPAT: DHAP acyltransferase; EK: ethanolamine kinase; EPT1: ethanolamine phosphotransferase; FAR1/2: fatty acyl-CoA reductase1/2; GPDH: glycerol-3-phosphate dehydrogenase; GPAT: glycerol-3-phosphate acyltransferase; LCLAT: acyl-CoA:lysocardiolipin acyltransferase; LPCAT: acyl-CoA:lysophosphatidylcholine acyltransferase; LPGAT: acyl.CoA:lysophosphatidylglycerol acyltransferase; PAP: phosphatidic acid phosphatase (lipin). PCYT1: phosphocholinetransferase; PCYT2: phosphoethanolaminetransferase; PEMT: SAM-phosphatidylethanolamine methyltransferase; PGPS: phosphatidylglycerophosphate synthase; PGPP: phosphatidylglycerophosphate phosphatase; PIS: phosphatidylinositol synthase; PISD: phosphatidylserine decarboxylase; PLA1/2: phospholipase A1/2; PLC: phospholipase C; PSS1/2: phosphatidylserine synthase 1/2; TGL: triacylglycerol lipase. Enzymes (**B**): ACER: alkaline ceramidase; CERK: ceramide kinase; CERS: Ceramide synthase; DES: sphingolipid 4-desaturase; GCST: galactosylceramide sulfotransferase; KDSR: 3-ketodihydrosphingosine reductase; PAP: phosphatidic acid phosphatase; SCPT: sphingosine cholinephosphotransferase; SmaseD: sphingomyelinase D; SMS: sphingomyelin synthase; SPT: serine:palmitoyltransferase; UGCG: ceramide glucosyltransferase; UGT8: ceramide galactosyltransferase.

Lipids play essential roles in a plethora of cellular and systemic processes, including cell compartmentalization, signaling and migration; fuel management; and protein trafficking and sorting [17,26,27]. To accomplish this, cells produce lipids with a vast structural complexity and diversity. A paradigmatic case is that of the membrane glycerophospholipids, as interconnected biochemical pathways, together with deacylation/reacylation processes, generate hundreds of molecular variants in terms of polar head groups (that determine the lipid class) and hydrophobic acyl chains (that determine the individual molecular species within a class). Schemes of core glycerophospholipid and sphingolipid biosynthetic pathways can be seen in Figure 1. This metabolic strategy provides a competitive advantage in nature, yielding a dynamic scaffold for cell membranes that allow cells to respond to changes in homeostasis and environmental cues. The response, however, may be short-lived. When it comes to the discovery of lipid biomarkers in tumor cells, a fundamental question is whether the expression of individual lipids that are formed through the pathways downstream of oncogenic signals (lipogenesis and others) can be measured and if they differ consistently from those of cells undergoing normal physiological turnover.

Mass spectrometry (MS)-based lipidomics offer a unique opportunity to study and screen hundreds of lipid species simultaneously, making it well-suited to lipid biomarkers discovery [28]. However, the structural complexity of lipids mentioned above and the numerous isomeric and isobaric species harboring the same backbone and acyl chains pose a major challenge to their identification. To the best of our knowledge, there have been few studies that have attempted to characterize the lipid profiles in melanocytes. Nano-electrospray ionization (ESI) chip-based direct infusion MS (DIMS), an analytical method without prior chromatographic separation of the lipid classes, was used to identify 65 lipids in the 500–900 *m*/*z* range expressed in HEMn-LP melanocytes and in two melanoma cell lines with low (A375) and high (A2058) metastatic potential [29]. When combined with a metabolomics approach, a partial least squares (PLS) projection to latent structure regression (PLSR) prediction model was developed to define a panel of six lipid species and 10 metabolites that differentiated melanocytes from melanoma cells with different metastatic potential. Prior to this, only the total lipid content and composition of certain melanoma cell lines had been examined [30]. On the other hand, we addressed the in situ classification of architectural features of benign intradermal melanocytic nevus lesions using imaging MS (IMS), developing a lipid expression-based algorithm that discriminates epidermis, dermis and melanocytes [31]. Consistent with the metabolic heterogeneity of melanoma cells within a single lesion [2], our IMS study also revealed distinct lipid phenotypes in melanocyte subpopulations within a single nevus, hinting that limited sample size (cell lines or nevus samples) may provide too narrow a window to obtain valuable biological information. Moreover, there are no lipidomic analyses of isolated benign human nevi melanocytes to date.

Nowadays, tandem MS preceded by ultrahigh performance liquid chromatography (UHPLC) separation of lipid classes is the method of choice in untargeted lipidomics [32]. This approach greatly enhances the accuracy and the sensitivity to identify low abundance lipids. Given the limitations mentioned above, here, we attempted to achieve extended coverage of the human melanocyte lipidome and test the hypothesis that a particular lipid fingerprint could discern benign and malignant melanocytes as well as melanoma cells with different metastatic potential. As such, we conducted untargeted UHPLC-MS^E^–based lipidomics on a panel of human epidermal melanocytes (HeM) and nevus melanocytes (NM), as well as on primary (PM) and metastatic (MM) melanoma cells grown in vitro (n = 4–10 per group). This study demonstrated that melanocytes exhibit a lipid phenotype characteristic of their biological status. Specifically, we identified a set of lipid metabolites that segregate epidermal and nevus melanocytes from either of the two melanoma groups, in which sphingomyelins and ether lipids play a fundamental role.

## 2. Results

### 2.1. Global Lipidomic Analysis of Human Melanocytic Cells

Lipidomic profiling of non-neoplastic and neoplastic melanocytic cells using our UHPLC-MS^E^ analytical platform enabled 209 lipid species to be identified and quantified in all the tested samples. The full list of lipid species and their corresponding peak markers and intensities can be seen in Table S1 and are summarized in Table 1. These metabolites belong to 16 classes from five lipid categories: sphingolipids—sphingomyelin (SM), ceramide (Cer) and hexosylceramide (HexCer), sterol lipids—cholesteryl ester (CE), glycerolipids—triglycerides (TG) and diglycerides (DG), glycerophospholipids—phosphatidylcoline (PC), lysoPC (LPC), phosphatidylethanolamine (PE), lysoPE (LPE), phosphatidylserine (PS), phosphatidylglycerol (PG), phosphatidylinositol (PI) and ether phospholipids PC(O/P) and PE(O/P) and free fatty acids (FA). For SM, HexCer, CE, TG, DG, PC, LPC and PC(O/P) the most intense ion was detected in positive polarity either as the protonated molecular ion or as the sodium or [M+NH4]^+^ adducts, whereas PE, LPE, PE(O/P), PS, PG, PI and FA were observed in negative ion mode as [M-H]^-^. In addition, our platform identified different Cer species in the two ionization modes, whereas, due to the low ionization capacity of sterols in ESI, only the neutral cholesteryl esters were recorded. In terms of the structural variability, storage TG exhibited by far the highest diversity, with 51 species identified, followed by FA and PC with 24 and 22 molecular variants, and PE plasmalogen and SM with 18 and 17 species, respectively. 

### 2.2. Different Lipid Signatures Feature Nonneoplastic and Neoplastic Melanocytic Cells

**Figure 2:**
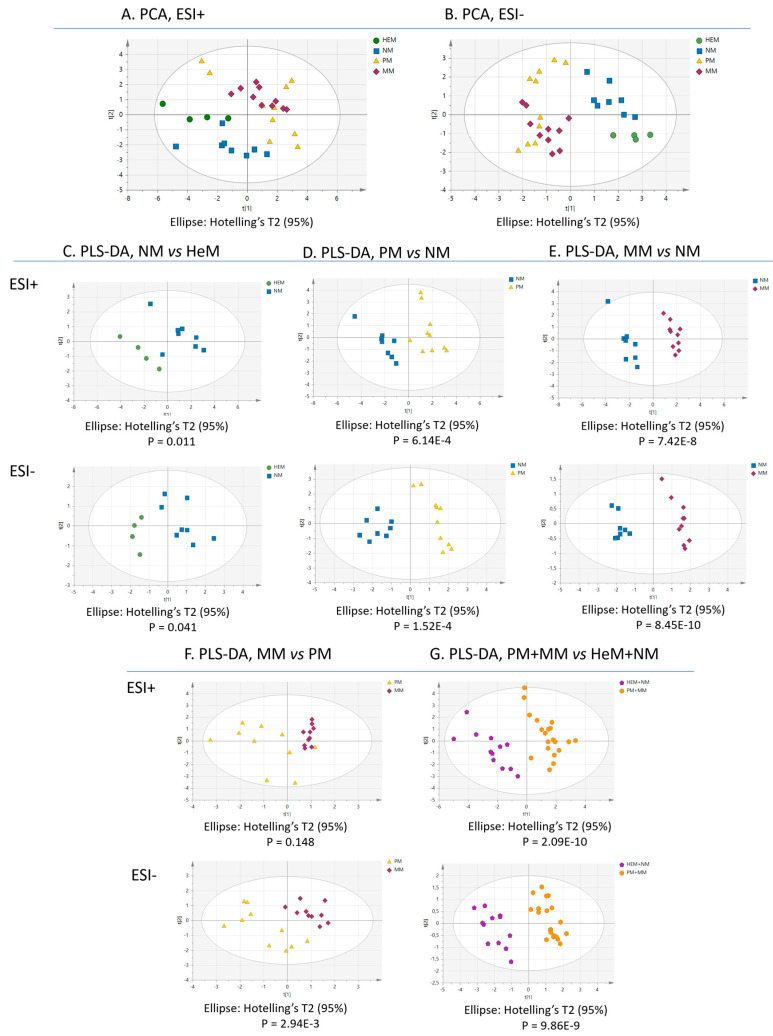
Multivariate statistical analysis of melanocytic cell lipidomes clearly discriminates benign from malignant cells. The principal component analysis (PCA) score plots of lipidomes obtained in the positive (**A**) and the negative (**B**) ion modes of UHPLC-MS^E^ are illustrated according to the independent cell samples (n = 4–10). Green, blue, yellow and red symbols indicate human epidermal melanocytes (HeM), nevus melanocytes (NM), primary melanoma (PM) and metastatic melanoma (MM), respectively. Binary comparisons constructed through partial least square-discriminant analysis (PLS-DA): (**C**) NM vs. HeM, (**D**) PM vs. NM, (**E**) MM vs. NM, (**F**) MM vs. PM, (**G**) melanoma (PM+MM, orange) vs. melanocyte (HeM+NM, blue).

To investigate the similarity of the lipid profiles in the samples, we applied multivariate statistical analysis to the data. The values for the quality parameters R2 (goodness of fit) and Q2 (ability of prediction) of the principal component analysis (PCA) plots (Figure 2A,B) indicate that the distinctive lipid expression was sufficient to group the samples according to their biology. All benign melanocytes were grouped in the same cluster and melanomas, in a different one. Nonetheless, the algorithm did not clearly discriminate melanoma cells according to their metastatic potential and some overlap between HeM and NM was evident in the positive-ion mode. All binary comparisons in the partial least square discriminant analysis (PLS-DA) produced a net group separation (NM vs. HeM, PM vs. HeM, MM vs. HeM, PM vs. NM, MM vs. NM, MM vs. PM), indicating that each cell type has a characteristic lipid profile in both ESI+ and ESI-. The results of this supervised analysis mimic what might be a canonical progressive transformation of epidermal melanocytes (Figure 2C–G). As shown, the differences between lipid patterns were striking in some cases, such as between PM and NM or MM and NM (Figure 2D,E), while they were subtle in others, such as between MM and PM (Figure 2F). PLS-DA further confirmed that the lipidome of melanocytes, taken as a single group (HeM+NM), differed markedly from the “malignant lipidome” harbored by PM-and-MM (Figure 2G). 

To validate the PLS-DA prediction models, we conducted permutation tests, recording the cumulative values of R2 and Q2 for 999 random rearrangements of the Y variables and thereafter, reconstructing the models (Table 2). The negative intersection of the Q2Y line of tendency obtained for all the models demonstrated that the PLS-DA data are well adjusted and the clustering was not by chance. An additional test, using *p*-values obtained from CV-ANOVA (Analysis of Variance testing of Cross-Validated predictive residuals) also confirmed the validity of the models, except for the MM vs. PM comparison in ESI+. Hence, the R2, Q2, permutation Q2 and *p*-values (Table 2) indicated that the models established reliably discriminate the cell groups based on their lipid fingerprint, except for the lipidomes of MM and PM in ESI+ that may not be sufficiently distinct to be clustered separately by this approach.

### 2.3. Changes in the Relative Abundance of Particular Lipid Species Reflect the Melanocyte Biology

**Figure 3:**
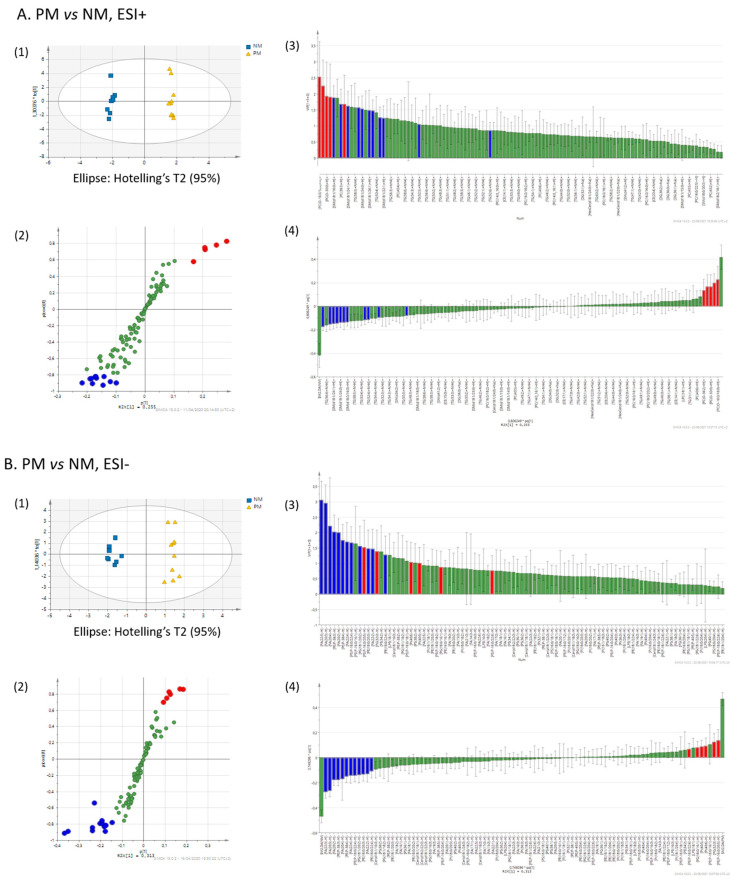
Representative selection of discriminant lipids by OPLS-DA. The lipidomes of primary melanoma (PM) and nevus melanocytes (NM) obtained in ESI+ (**A**) and ESI- (**B**) were evaluated by OPLS-DA for candidate biomarker selection. In the score plots of the two main components (1), discriminant lipids were visualized as S-plots (2), VIP plots (3), and loading plots calculated by the jack-knife algorithm. Red and blue denote the variables that are more and less abundant in PM than in NM, respectively.

To extract putative lipid biomarkers of melanocytes in different states, we performed a multivariate orthogonal partial least square-discriminant analysis (OPLS-DA) of the lipid fingerprints, employing the following threshold parameters: fold-change > 1.3 or < 0.7 (ion peak intensity ratio), coefficient of variation < 30%, ANOVA *p* < 0.05, variable importance in the projection (VIP) > 1.0. This approach enabled the top discriminant lipids to be selected (Table 3). An example of the selection of discriminant lipids between PM and NM is shown in Figure 3A,B. 

A set of 53 lipid metabolites showed the strongest discriminatory power across groups (about 25 % of the total number of lipid species identified), including PC(O), PE(P), PG, PI, PE, PC, SM, TG and FA (100, 39, 62, 60, 17, 5, 47, 20 and 25 % of the molecules in their respective classes). The identities, the detection parameters and the typical changes of these species are reported in Table 3. Intergroup differences were confirmed by several univariate statistical analyses. In particular, the *p*-values from the ANOVA with post hoc testing (Levene and Tukey or Games–Howell tests) are shown (Table 3, see asterisks).

Volcano plots and heatmap clustering were performed applying a fold change >2 and a *p*-value ≤ 0.05 by Student’s t-test using Benjamini–Hochberg as a multiple testing correction (Figure S1). Having less stringent discriminant statistical power than the OPLS-DA thresholds we assigned, univariate analyses expanded the list of potential biomarkers. Typically, new molecular species were added within the same lipid classes defined by OPLS-DA (Table 3).

**Table 3:** Discriminant lipid metabolites of melanocyte biology extracted by OPLS-DA.

Lipid	Adduct Characterized	*m/z*	RT	NM vs. HeM	PM vs. HeM	MM vs. HeM	PM vs. NM	MM vs. NM	MM vs. PM	PM+MM vs. HeM+NM
PC O-30:0	[PC(O-30:0)+H]^+^	692.5601	4.39	down ***		up ***	up **	up ***		up ***
PC O-32:1	[PC(O-32:1)+H]^+^	718.5743	4.48	down **		up **	up ***	up ***		up ***
PC O-16:0/16:0	[PC(O-16:0/16:0)+H]^+^	720.5921	5.02	down ***		up ***	up ***	up ***		up ***
PC O-16:0/18:1	[PC(O-16:0/18:1)+H]^+^	746.6087	5.08	down ***		up ***	up ***	up ***		up ***
PC O-36:2	[PC(O-36:2)+H]^+^	772.6240	5.13	down ***		up ***	up *	up ***		up **
PE P-16:0/16:1	[PE(P-16:0/16:1)-H]^−^	672.4963	4.59					up		
PE P-16:0/20:5	[PE(P-16:0/20:5)-H]^−^	720.4969	4.20		up ***	up ***	up ***	up ***		up ***
PE P-16:0/22:6	[PE(P-16:0/22:6)-H]^−^	746.5122	4.42			up ***	up **	up ***	up ***	up ***
PE P-38:4	[PE(P-38:4)-H]^−^	750.5421	5.17		down *	down	down ***	down ***		down ***
PE P-38:3	[PE(P-38:3)-H]^−^	752.5569	5.45		down	down	down ***	down **		down ***
PE P-18:0/22:6	[PE(P-18:0/22:6)-H]^−^	774.5456	5.03			up ***		up ***	up ***	
PE P-18:0/22:4	[PE(P-18:0/22:4)-H]^−^	778.5733	5.56				down **	down *		down **
PG 32:0	[PG(32:0)-H]^−^	721.4996	4.21		up ***	up ***	up ***	up ***		up ***
PG 18:0/16:1	[PG(18:0/16:1)-H]^−^	747.5139	4.47			up ***	up **	up ***	up ***	up ***
PG 36:1	[PG(36:1)-H]^−^	775.5451	5.04			up ***		up ***	up ***	
PG 18:1/18:2	[PG(18:1/18:2)-H]^−^	771.5170	3.13		down ***	down **				
PG 18:1/20:2	[PG(18:1/20:2)-H]^−^	799.5449	3.62		down ***	down ***	down ***		up ***	down ***
PI 32:1	[PI(32:1)-H]^−^	807.5026	3.10					up *		
PI 16:1/18:0	[PI(16:1/18:0)-H]^−^	835.5326	3.67					up *		
PI 16:0/20:4	[PI(16:0/20:4)-H]^−^	857.5176	3.14					up ***		
PI 18:0/18:2	[PI(18:0/18:2)-H]^−^	861.5506	3.75					up ***		
PI 16:0/20:1	[PI(16:0/20:1)-H]^−^	863.5633	4.26					up *		
PI 18:1/20:4	[PI(18:1/20:4)-H]^−^	883.5350	3.24					up ***		
PI 18:0/20:2	[PI(18:0/20:2)-H]^−^	889.5792	4.31					up ***	up **	
PI 40:6	[PI(40:6)-H]^−^	909.5487	3.59		up ***	up ***	up ***	up ***		up ***
PI 40:5	[PI(40:5)-H]^−^	911.5645	3.76			up **	up ***	up ***		up ***
PE 18:0/20:3	[PE(18:0/20:3)-H]^−^	768.5539	5.25	down	down *	down *				
PE 18:0/22:4	[PE(18:0/22:4)-H]^−^	794.5681	5.25		down ***	down ***	down ***	down *		down ***
PC 38:3	[PC(38:3)+H]^+^	812.6182	4.92		down ***		down ***	down ***		down ***
SM d18:1/14:0	[SM(d18:1/14:0)+H]^+^	677.5562	3.38			down **		down ***		
SM d18:1/16:0	[SM(d18:1/16:0)+H]^+^	703.5790	3.93		down ***	down ***	down ***	down ***		down ***
SM d18:0/16:0	[SM(d18:0/16:0)+H]^+^	705.5837	3.97		down ***	down ***	down ***	down ***		down ***
SM d18:1/22:1	[SM(d18:1/22:1)+H]^+^	785.6613	5.23	down *	down ***	down ***				
SM d18:1/22:0	[SM(d18:1/22:0)+H]^+^	787.6701	5.76		down ***	down	down ***	down **		down ***
SM d18:1/24:1	[SM(d18:1/24:1)+H]^+^	813.6875	5.74		down ***		down ***	down ***		down ***
SM d18:1/24:0	[SM(d18:1/24:0)+H]^+^	815.7000	6.31		down	down	down ***	down **		down **
SM d18:1/26:1	[SM(d18:1/26:1)+H]^+^	841.7153	6.27	up			down ***	down **		
TG 50:4	[TG(50:4)+NH4]^+^	844.7390	7.99		down	down		down *		down ***
TG 52:6	[TG(52:6)+NH4]^+^	868.7380	7.76		down ***	down ***				
TG 52:5	[TG(52:5)+NH4]^+^	870.7568	7.99		down ***		down ***			
TG 52:4	[TG(52:4)+NH4]^+^	872.7720	8.22		down ***		down ***			down ***
TG 54:5	[TG(54:5)+NH4]^+^	898.7851	8.24		down ***	down ***	down ***			down ***
TG 54:4	[TG(54:4)+NH4]^+^	900.8043	8.47		down ***	down ***				down ***
TG 56:5	[TG(56:5)+NH4]^+^	926.8162	8.55		down ***	down ***				down ***
TG 56:4	[TG(56:4)+NH4]^+^	928.8324	8.73		down ***	down ***				
TG 58:5	[TG(58:5)+NH4]^+^	954.8464	8.77		down **	down ***		down **		down ***
TG 58:4	[TG(58:4)+NH4]^+^	956.8638	8.92		down ***	down ***		down **		down ***
FA 20:4	[FA(20:4)-H]^−^	303.2335	1.24	down	down	down *				
FA 20:3	[FA(20:3)-H]^−^	305.2482	1.52		down ***	down ***				
FA 20:2	[FA(20:2)-H]^−^	307.2624	1.82	down	down ***	down ***				
FA 22:4	[FA(22:4)-H]^−^	331.2637	1.62		down ***	down ***	down ***	down ***		down ***
FA 22:3	[FA(22:3)-H]^−^	333.2795	2.01		down ***	down ***	down ***	down ***		down ***
FA 22:2	[FA(22:2)-H]^−^	335.2944	2.43		down ***	down ***	down ***	down **		down ***

The asterisks indicate significant intergroup differences following one-way ANOVA and post-hoc testing with Tukey (*p* ≥ 0.05) or Games–Howell’s (*p* ≤ 0.05) after assessing the equivalence of the variances by Levene’s test: * *p* ≤ 0.05, ** *p* ≤ 0.01, *** *p* ≤ 0.001. RT, retention time; *m/z*, mass-to-charge ratio; HeM, human epidermal melanocytes; NM, nevus melanocytes; PM, primary melanoma; MM, metastatic melanoma; PC, phosphatidylcholine; PC-O, ether PC; PE, phosphatidylethanolamine; PE-P, vinyl ether PE; PG, phosphatidylglycerol; PI, phosphatidylinositol; SM, sphingomyelin; TG, triglyceride; FA, fatty acid.

From a biological perspective, it is worth noting that our findings revealed that 1—sphingomyelins and glycerophospholipids contributed most significantly to the phenotypic diversity of melanocytes; 2—the changes in glycerophospholipids were highly selective, showing functional group, FA and FA-linkage type specificity; 3—lipid fingerprints strongly differentiated between neoplastic and non-neoplastic melanocytes; 4—there were significant differences, although less numerous, in lipid species between melanoma cells with different metastatic potential and between epidermal and benign nevus melanocytes.

The dominating characteristic of melanoma cells compared to normal melanocytes was the elevation of ether-type PC (100% of the total number of molecules within this class) and the dramatic decrease in SM, involving 8 of the 17 molecules identified that represented around 80% of the total SM intensity. An incorrect pattern of PE plasmalogens and PG was also found in melanoma, characterized by the presence of some strongly expressed species while others were dampened intensely, along with a decrease in some TG and FA species. The intensity of lipid species eliciting opposing trends in the experimental groups is exemplified by the ethyl ethers PC O-16:0/16:0 and PC O-16:0/18:1, which are expressed more strongly in malignant cells (Figure S2A), and by SM d18:1/16:0 and PE P-18:0/22:4, that were particularly abundant in melanocytes (Figure S2B). Figure S2 also provides clear examples of the heterogeneity in lipid expression across the cell lines analyzed, which were used to model a condition, as also seen for colon cancer cell lines [33]. 

An additional finding that may have signaling consequences is the increased relative abundance of PI species in metastatic melanoma cells in respect to normal melanocytes (most markedly versus nevi melanocytes).

**Figure 4:**
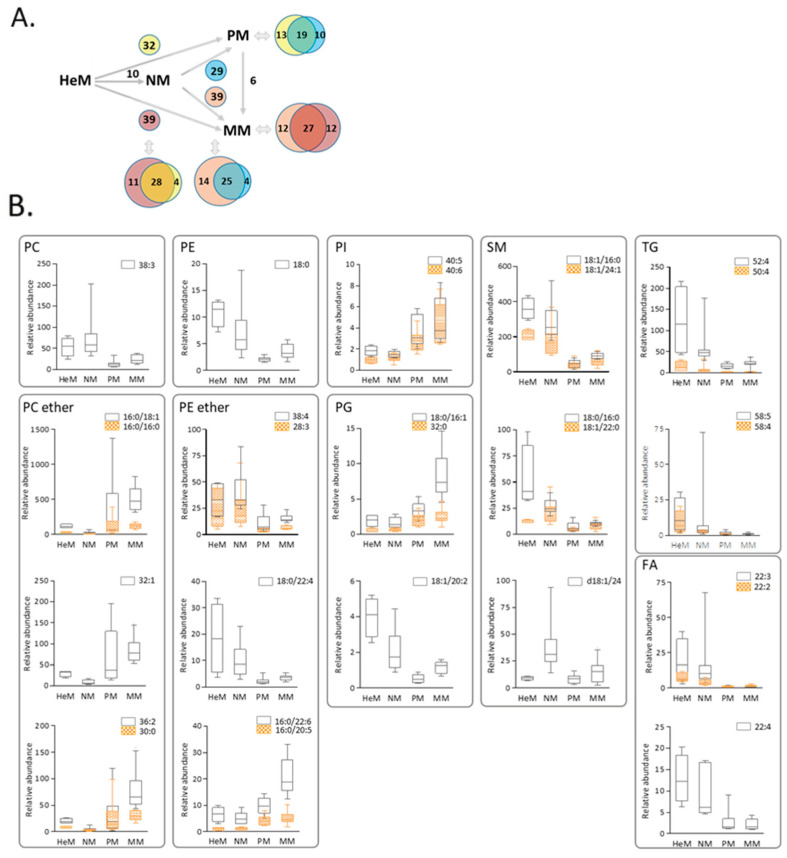
Primary and metastatic melanoma exhibit common and specific changes in lipid signatures compared to epidermal or nevus melanocytes. (**A**) Changes in lipid species in PM compared to HeM (yellow) or NM (blue), and in MM compared to HeM (purple) or NM (pink). (**B**) Box–Whisker plots of the 32 lipids with the strongest discriminatory power of the neoplastic phenotypes. The whiskers in the box plots represent the 90% and 10% values. HeM, human epidermal melanocytes; NM, nevi melanocytes; PM, primary melanoma; MM, metastatic melanoma. PC, phosphatidylcholine; PE, phosphatidylethanolamine; PI, phosphatidylinositol; PG, phosphatidylglycerol; SM, sphingomyelin; TG, triglyceride; FA, fatty acid.

It is important to underline that similar and specific differences were observed in PM and MM lipidomes to those observed in epidermal and nevi melanocytes (see Venn diagrams in Figure 4B). For instance, similar changes in nineteen lipids, a decrease in 16 and an increase in three molecular species differentiated primary melanoma from both epidermal and nevus melanocytes, suggesting that this common phenotypic remodeling may be associated with tumor initiation and the maintenance of primary melanoma. The 32 candidate biomarkers of malignancy extracted from the PM+MM vs. HeM+NM analysis were examined in more detail (Figure 4B). Discriminant lipids belong to nine classes: five choline ether and five ethanolamine ether lipids, 1 PC, 1 PE, 2 PI, 3 PG, 5 SM, 3 FA and 7 TG (only four are plotted). Interestingly, there was a selective and striking shift in species from a diacyl-type PC and PE to PC ether and PE plasmalogens, and in particular to low unsaturated PC ether species and to plasmalogens presenting 20:5 (eicosapentaenoic acid, EPA) or 22:6 (docosahexaenoic acid, DHA) at the *sn*-2 position. In addition, low levels of most SM species and of 20C and 22C FA were part of the common composite of malignant melanocytes, in conjunction with aberrant PG expression, a lipid class unique to mitochondrial membranes [34].

As mentioned earlier, a literature search failed to retrieve any lipidomic data from melanocytes isolated from clinically diagnosed benign nevus. By contrast, here we defined a detailed lipidome for the NM and HeM phenotypes (Table S1) and compared their profiles (Figure 2C and Figure 4, Table 3). Apart from a switch in a pair of SM species, an interesting finding was the decline of all PC ethers to levels below 25% of those recorded in HeM.

The melanocyte lipid landscape was further analyzed by assessing whether the variations in species abundance affect the relative expression of the total lipid classes. To study this, we assessed the sums of the intensities of all the lipid species within each class (for the source data, see Table S1). Statistically significant differences were observed among some classes by ANOVA and post-hoc testing (Levene and Tukey or Games–Howell) (Figure S3A–C) that revealed a general trend towards expanded signaling lipids PI, PG, Cer and DG in metastatic melanoma as opposed with primary melanoma. Once again, choline ether phospholipids were expanded while SM plummeted in neoplastic cells relative to non-neoplastic melanocytes. Therefore, abnormal signaling pathways may be envisaged in melanoma cells with high metastatic potential, in addition to remodeling of ether glycerophospholipids and sphingolipid metabolism in melanoma cells irrespective of their metastatic potential.

## 3. Discussion

Different models have been proposed to explain melanoma progression and one of the most popular, the Clark model, considers the melanoma development to be a linear and stepwise transformation: benign nevi, dysplastic nevi, primary melanoma in the radial growth phase, primary melanoma in the vertical growth phase and metastatic melanoma. However, many melanoma tumors may not follow such an orderly progression. Nevertheless, over the past two decades, the biochemical changes that accompany these transitions have begun to be defined, although the molecular mechanisms that distinguish the pathological and benign transformations of melanocytes have yet to be fully explored. As such, it remains unclear whether these differences can be targeted therapeutically. 

Here we studied HeM, NM, PM and MM cells through untargeted UHPLC-MS^E^-based lipidomics, defining different lipid signatures related to the benign and malignant transformation of melanocytes. We identified significant changes to 53 lipid species that tell us more about how melanoma grows and spreads and how nevus melanocytes do not share the perturbations in the lipid metabolism of melanoma cells. In fact, the most prominent differences across groups were observed between nevus melanocytes and primary and metastatic melanoma cells. We further identified distinctive traits between NM and HeM involving ether PC species, between MM and PM involving six lipids and between malignant melanoma cells and normal melanocytes that revealed 32 biomarker candidates. 

A first remarkable observation is the selective accumulation of ether PC lipids in primary and metastatic melanoma, whereas, in strong contrast, nevus melanocytes were depleted in PC ethers relative to epidermal melanocytes. Thus, ether PC levels can distinguish a benign from a malignant transformed cell, discriminating the melanocyte status. 

Ether glycerophospholipids represent an important group of membrane phospholipids in which the hydrocarbon chain is linked by an ether (O) or vinyl ether (P) bond at the *sn*-1 position of the glycerol backbone. In mammals, ether phospholipids are initially synthesized in peroxisomes (driven by the FAR1 and FAR2 enzymes that catalyze the required reduction of a fatty acid to a fatty alcohol) and processed in the endoplasmic reticulum (ER) [35,36]. Ether phospholipids are known to be involved in signaling, the export of GPI-anchored proteins from the ER and the resistance to insults like oxidative stress [35,37,38,39,40] and ferroptosis [41]. The increased generation of reactive oxygen species (ROS) due to an enhanced metabolic activity is a common stressor to which cancer cells must adapt and buffer. This increased generation of ROS can play a dual role in the cancer phenotype: a tumorigenic role promoting growth and genomic instability or be toxic and induce cell death (reviewed in [10]). However, in non-neoplastic cells, ROS promotes senescence and apoptosis. Thus, it is conceivable that peroxisome-driven enrichment in ether PC would confer a survival advantage during tumorigenesis and metastasis, providing melanoma cells strong protection due to their antioxidant-scavenging capacity upregulation. By contrast, the drop of ether lipids in normal nevus melanocytes would render cells to a state that makes them vulnerable to senescence and apoptosis. Untangling the molecular determinants of ether PC depletion in nevus melanocytes, a finding first reported here, will likely offer additional insight to devise strategies that target redox homeostasis in melanoma. 

Notably, PE plasmalogens did not follow the ether PC trends. Instead, exchanges of one PE plasmalogen species for another were observed among different groups, suggesting that a second non-peroxisomal level of regulation may exist. Interestingly, two DHA-containing plasmalogens, PE P-16:0/22:6 and PE P-18:0/22:6, were identified as potential biomarkers of metastasis. In plasmalogens, the vinyl ether bond is formed at the ER from the corresponding ether PE precursor in a recently discovered, TMEM189-catalyzed reaction [42]. It is tempting to speculate that TMEM189 expression or function may be driven by specific signals with implications in metastasis. This is consistent with findings of a recent expression profile analysis of TMEM189 in the cancer databases that revealed that cancer patients expressing higher levels of TMEM189 have a significantly shorter overall survival compared to patients bearing tumors expressing lower levels of TMEM189 [41].

A second remarkable feature is that SM, both as a class and in terms of the major SM species, are underrepresented in neoplastic cells, with similar levels in primary and metastatic melanoma. Because SMs are essential components of cell membranes, this may be functionally relevant. Besides influencing membrane fluidity, SMs are known to regulate critical aspects of cell division, proliferation and chemotaxis, events that could contribute to cancer development (we refer the reader to two recent excellent reviews [43,44]). SM forms membrane microdomain platforms—along with cholesterol and plasmalogens—to transduce transmembrane signals with wide implications in apoptosis, proliferation, differentiation and inflammation, among other functions [43,45,46,47]. The enzymes ultimately responsible for SM synthesis, SM synthase (SMS) 1 and 2, transfer a phosphocholine moiety from PC to ceramide, forming SM and diglycerides. Defects in SM synthesis may therefore not only have direct but also indirect consequences, bringing together SM deficiencies with PC, ceramide and DG metabolism, lipids with long recognized key specific structural and signaling roles. The deficiency of SMS activity was reported to enhance cell migration in an SMS1-SMS2 double-knockout cellular model [45]. However, we did not find significant differences in SM levels between primary and metastatic phenotypes, whereby underrepresentation of both SM species and SM class in melanoma is strongly associated with malignancy but not with metastasis, at least in the collection of melanoma cell lines studied here. It is of interest to bear in mind that, although altered SM levels have been reported in numerous cancers, the specific changes seem to depend on the type of cancer. For instance, increased SM levels were found in human hepatocellular carcinoma [48] but conversely, and in line with the lipid changes reported here, SM levels dropped in colorectal [49] and breast cancer [50] cell lines as well as in prostate cancer tissue [51] and clear cell renal cell carcinoma [16] relative to non-malignant tissues. The fact that SM synthesis is finely regulated not only at the SMS step but also at multiple levels involving the metabolism of other lipids and numerous protein-mediated inter-organelles transport systems [43] may underlie the different responses to particular oncogenic stimuli.

Kim et al. [29] identified five PI species and one PG that differentiated melanomas with low and high metastatic potential, as well as malignant melanoma and normal melanocytes. Here, we found that two PI species containing polyunsaturated fatty acids (40:4 and 40:6) and three PG harboring low unsaturated acyl chains also discriminate melanoma from normal melanocytes and seven more PI species distinguish MM from NM. In addition, we found increased PI class levels in MM relative to PM cells. Except for one PI, the identities of the species were not the same, possibly due to the differences in the experimental approach, both in terms of the number of cell lines analyzed and the analytical procedures used. In the present study, we employed UHPLC-MS^E^-based untargeted lipidomics, which greatly enhances sensitivity for low abundance lipids and the accuracy of identification. By contrast, Kim et al. used a DIMS-based lipidomics assay [29], which is known to be prone to ion suppression due to the absence of a previous separation step, also compromising identification. Previous comparative studies on the ability of DIMS and HPLC-MS to identify renal cancer biomarkers also concluded that the latter was able to identify a larger number of biomarkers and found differences in the identities [52]. Moreover, as intratumor heterogeneity is inherent to tumor development, we examined a robust number of cell lines to explore what could be considered a “consensus panel of lipid components,” i.e., lipids consistently expressed in melanocytic cells. Although it may appear that there are discrepancies between these two studies, one similar message emerges: metastatic melanoma herein and highly metastatic melanoma in Kim et al.’s [29] study are enriched in PI lipids. Although PI accounts for only a small fraction of the plasma membrane lipids, it plays a critical role in the cell, especially in signal transduction [27].

From a biological standpoint, a general consideration to have in mind is that changes in lipid species may be viewed as either metabolic compensation, with or without apparent impact on cell function, or in terms of the provision or decline of a particular lipid responsible for or involved in a given biological process. As shown in Figure 1, a huge interconnection of the glycerophospholipid biosynthetic pathways takes place in the ER, with the exception of some reactions that occur in peroxisomes and mitochondria and that are involved in the synthesis of ether lipids and phosphatidylglycerol-containing lipids, respectively. Moreover, while the role of most lipid classes is well established, limited information is available on the role of individual species other than the influence of the length and saturation of acyl chain composition on the biophysical properties of membranes and the response to proinflammatory signals. Fortunately, this information is being expanded by the application of MS to the analysis of lipids [27]

Finally, some caution should be exerted when drawing inferences from this study as cell lines do not model all facets of melanocytes/melanoma in situ. One of the most apparent limitations is that the FA profile of cells in culture often differs markedly from that of cells in a tissue [53], where cells are exposed to a changing variety of nutrients in their environment, oxygen availability fluctuates and extracellular matrix interactions occur. Furthermore, melanoma shows impressive metabolic plasticity and might switch between coexisting phenotypes during tumorigenesis. Here, we reveal lipid species and surrogate metabolic processes that differ substantially between cells cultured in vitro, paving the way to identify potential biomarkers and therapeutic opportunities that target metabolic networks in melanoma. However, validation of these data in vivo will be essential using IMS of lipids or other spatial phenotypic techniques on biopsies or true samples of patient-derived melanomas and nevi melanocytes. 

In conclusion, through a lipidomics analysis, we reveal alterations to the lipid profiles of melanocytes that appear to be related to their benign and malignant transformation. We found 32 putative melanoma lipid biomarkers; one of them had already been identified previously [29], six lipid species were related to metastasis and 10 discriminate nevus melanocytes from epidermal melanocytes. Unraveling the lipidomic profile of nevus melanocytes and comparing it to that of primary melanoma cells is of utmost interest to decipher the lipid contribution in the realm of benign/malignant transformation.

## 4. Materials and Methods

### 4.1. Subjects, Cell Culture and Sample Preparation for Lipidomic Analysis

Primary melanoma (PM: SK-MEL-28, SK-MEL-31, G-361, MEL-HO and A375), metastatic melanoma (MM: RPMI-7951, Hs294T, A2058, SK-MEL-3 and Colo-800) and human epidermal melanocyte (HeM: HEMn-LP, HEMn-MP, and HEMn-DP) cell lines were obtained from the American Type Culture Collection (ATCC, Manassas, VA, USA), Thermo Fisher Scientific (Waltham, MA, USA) or Innoprot SL (Derio, Spain). Melanocytes from histologically confirmed, benign melanocytic nevi lesions (n = 8) were isolated [54] and characterized according to our previously reported procedure [55]. Nevi were collected by the Basque Biobank for Research-OEHUN (http://www.biobancovasco.org, accessed on 24 October 2021), having previously obtained signed informed consent from donors. All the experiments carried out in this study were approved by the Euskadi Ethics Committee (Oncoimage, 14/10). 

Melanoma cells were propagated in EMEM (ATCC), DMEM (Sigma-Aldrich, Burlington, MA, USA) or McCoy’s 5A medium (Life Technologies, Carlsbad, CA, USA) supplemented with 2 mM L-glutamine (Sigma-Aldrich), or in RPMI 1640-Glutamax™ (Life Technologies). The different culture media were supplemented with 10% FBS (GE Healthcare, Chicago, IL, USA), 100 U/ml penicillin and 100 mg/ml streptomycin (Life Technologies), according to the manufacturer’s instructions. Primary human melanocytes were cultured in Medium 254 supplemented with 1X human melanocyte growth supplement (Life Technologies) in the absence of antibiotics. Cells were cultured at 37 °C with 5% CO_2_ and 95% humidity.

Lipid extraction was performed as described previously [56]. Briefly, cell pellets were first rinsed twice and homogenized in 0.5–1 mL PBS using a Polytron homogenizer (Kinematica AG, Malters, Switzerland), and the protein concentration of the homogenate was determined (Bio-Rad protein assay kit, Thermo Fisher Scientific). Lipids were extracted twice from homogenates (1 mg protein) with chloroform and methanol and the lower lipid-rich chloroform phases were combined in a new tube and solvents evaporated under vacuum (Savant SpeedVac concentrator, Thermo Fisher Scientific). The dried extracts were reconstituted in chloroform:methanol (2:1, *v*/*v*), transferred to liquid chromatography vials and the solvents dried. Finally, the extracts were stored at −80 °C under an N_2_ atmosphere prior to their injection into the UHPLC-MS^E^ system. Equal aliquots of each sample were pooled to make quality control samples for periodic analysis of repeatability.

### 4.2. UHPLC-MS^E^ Analysis

Global lipidomic profiles were determined by tandem MS using an ESI in the negative (−) and positive (+) ion mode after the separation of lipid classes by a reverse-phase UHPLC technique. The chromatographic separation was achieved on an ACQUITY UPLC^TM^ system from Waters (Milford, MA, USA), equipped with a binary solvent delivery pump, an autosampler and a column oven. A reverse-phase column (Acquity UHPLC HSS T3, 100 × 2.1 mm, 1.8 µm) and a pre-column (Acquity UHPLC HSS T3 1.8 µm VanGuard™), both from Waters, were used. The mobile phases and UHPLC settings are shown in Table S2, and the analysis was based on our previous report [57].

All UHPLC-MS^E^ data were acquired on a SYNAPT G2 HDMS, with a quadrupole time of flight (Q-ToF) configuration (Waters) equipped with an ESI source operating in positive and negative ion modes. The optimized settings are detailed in Table S2. The spectra were acquired over the 50–1200 *m/z* range and automatically corrected during acquisition using the lock mass with leucine encephalin solution (2 ng/µL) in acetonitrile:water (50:50, *v*/*v*) and 0.1% formic acid. The reference internal standards were introduced into the lock mass sprayer at a constant flow rate of 10 µL/min using an external pump. 

The UHPLC-MS^E^ data generated were extracted with MSE Data Viewer [58]. This software generates an exportable text file, which was used for lipid identification using SimLipid [59]. The identity of the compounds was elucidated using the low-collision energy MS^E^ spectrum in positive and negative modes to determine the MW, and the high-collision energy MS^E^ spectrum to elucidate other structural details. The mass tolerance window was set to 5 ppm for precursor and product ions. Annotation was reinforced by comparing theoretical to experimental MS/MS spectra (Figure S4) and assessment of the intensity of the carboxylate ion peaks corresponding to the fatty acyl chains on the *sn*-1 and *sn*-2 positions of the glycerol backbone (Figure S5).

The nomenclature and shorthand notation of lipid species was reported as recommended in the LIPIDMAPS update report [60]. Whenever possible, annotation is given at the *sn*-position (i.e.: PC 16:0/18:1), but FA positions may be unknown (PC 16:0_18:1) or unidentified (PC 34:1). The UHPLC-MS^E^ platform differentiated the ether and vinyl ether bonds of alkyls at the *sn*-1 position of the glycerol backbone from the diacyl GPL (represented by O- and P-, respectively). 

### 4.3. Data Processing and Statistical Analysis

Raw MS data, acquired with MassLynx 4.1 [61], were converted into NetCDF files [62] and processed [63]) to generate a table of time-aligned features, with the retention time (RT), *m/z* and intensity values for each sample. The Matched Filter detection algorithm was used for peak identification and the R-package CAMERA 1.22.0 [64] to exclude the detected isotopologues of molecular entities. The peak marker tables (comprising *m*/*z*-RT pairs and their corresponding intensities) were exported to SIMCA 15.0.2 [65] for multivariate statistical analysis and to SPSS 26 [66] and Mass Profiler Professional B14.8 [67] for univariate analysis. 

Differences in the lipid fingerprints among the groups were examined using multivariate statistical methods, including a principal components analysis (PCA) and partial least square and orthogonal partial least square-discriminant analysis (PLSA-DA and OPLS-DA, respectively). The data matrix of the original features was logarithmically transformed [log10(xij + 1)] to improve the normality and homoscedasticity, and centered using Mean Centering in the SIMCA software prior to performing the multivariate data analysis. We explored unsupervised PCA as an overview to look for trends and groupings and supervised PLS-DA to generate predictive models that were validated by means of permutation test and their *p*-values obtained from CV-ANOVA (Analysis of Variance testing of Cross-Validated predictive residuals). To select discriminant lipids, we explored the OPLS-DA models and further confirmed these using ANOVA and post-hoc testing with HSD-Tukey or Games–Howell after performing Levene’s test to assess the equivalence of variances of the groups (Games–Howell if *p* ≤ 0.05; HSD-Tukey if *p* ≥ 0.05).

We also carried out univariate studies using ANOVA analysis corrected with a post-hoc HSD Tukey or Games–Howell approach (see above) and applying a *p* ≤ 0.05 to determine the significant inter-group differences. Volcano plots and heatmaps were also obtained to identify inter-group differences in lipid composition, with a fold change >2 and *p* ≤ 0.05 by Student’s t-test using Benjamini–Hochberg FDR < 5% as a multiple testing correction. 

## 5. Patents

European patent No. EP21382988.0, “Method for the diagnosis of melanoma”, IMG Pharma Biotech, S.L. and Universidad del País Vasco/Euskal Herriko Unibertsitatea. Application date: 3 November 2021.

## Figures and Tables

**Table 1:** Lipid metabolites identified by lipidomics in non-neoplastic and neoplastic melanocytes. A total of 209 lipid species from 16 classes were identified in each of the samples analyzed in the positive and negative ion modes of UHPLC-MS^E^.

Category	Lipid Class	Abbreviation	Lipid Species (n)	Most Intense Adduct	Most Abundant Metabolite
Sphingolipids	Sphingomyelin	SM	17	[SM+H]+	SM d18:1/16:0
	Hexosylceramide	HexCer	3	[HexCer+Na]+	HexCer d18:1/24:1
	Ceramide	Cer	2	[Cer+Na]+	Cer d18:1/24:1
	Ceramide	Cer	6	[Cer-H]−	Cer d18:1/16:0
Sterol lipids	Cholesteryl ester	CE	6	[CE+NH_4_]+	CE 15:0
Glycerolipids	Triglyceride	TG	51	[TG+NH_4_]+	TG 52:3
	Diglyceride	DG	7	[DG+Na]+	DG 36:0
Glycerophospholipids	Phosphatidylcholine	PC	22	[PC+H]+	PC 16:0/18:1
	Lysophosphatidylcholine	LPC	1	[LPC+H]+	LPC 18:1
	Ether-PC	PC(O/P) ^1^	5	[PC(O)+H]+	PC O-16:0/18:1
	Phosphatidylethanolamine	PE	12	[PE-H]−	PE 18:0/18:1
	Lysophosphatidylethanolamine	LPE	4	[LPE-H]−	LPE 20:4
	Ether-PE	PE(O/P) ^1^	18	[PE(P)-H]−	PE P-16:0/18:1
	Phosphatidylserine	PS	8	[PS-H]−	PS 18:0/18:1
	Phosphatidylglycerol	PG	8	[PG-H]−	PG 16:0/18:0
	Phosphatidylinositol	PI	15	[PI-H]−	PI 18:0/20:4
Fatty acyls	Fatty acid	FA	24	[FA-H]−	FA 18:0

^1^ In the shorthand notation for ether species, O− and P− represent the ether and vinyl ether bonds of alkyls at the *sn*-1 position of the glycerol backbone, respectively.

**Table 2:** Validation of the PLS-DA models.

Model	Ionization Mode	Principal Components, Minimum ^1^	R2	Q2	Q2 Intercept	*p*-Value ^2^
NM vs. HeM	+	2	0.831	0.595	−0.218	1.07 × 10^−2^
	−	1	0.741	0.509	−0.147	4.10 × 10^−2^
PM vs. HeM	+	3	0.966	0.908	−0.399	6.14 × 10^−4^
	−	2	0.967	0.929	−0.346	4.54 × 10^−5^
MM vs. HeM	+	2	0.949	0.907	−0.246	1.28 × 10^−4^
	−	2	0.971	0.930	−0.280	3.15 × 10^−5^
PM vs. NM	+	3	0.976	0.919	−0.444	1.21 × 10^−4^
	−	3	0.968	0.870	−0.442	1.52 × 10^−4^
MM vs. NM	+	2	0.972	0.945	−0.345	7.42 × 10^−8^
	−	1	0.953	0.938	−0.262	8.45 × 10^−10^
MM vs. PM	+	1	0.516	0.201	−0.076	1.48 × 10^−1^
	−	4	0.965	0.825	−0.495	2.94 × 10^−3^
PM and MM vs. HeM and NM	+	3	0.944	0.879	−0.382	2.09 × 10^−10^
	−	4	0.967	0.891	−0.544	9.86 × 10^−9^

^1^ Minimum number of principal components required to explain the maximum variance. ^2^ The validity of the model according to that minimum number of principal components. HeM, human epidermal melanocytes; NM, nevus melanocytes; PM, primary melanoma; MM, metastatic melanoma.

## Data Availability

The data that support the findings of this study are available from the corresponding author upon reasonable request.

## Supplementary Materials

The following are available online at https://www.mdpi.com/article/10.3390/ijms222112061/s1: Table S1: list of lipid species identified by lipidomics in melanocytic cells; along with the relative intensity in each independent sample and the corresponding peak markers (mass to charge ratio (*m/z*) and retention time (RT) pairs); Table S2: ultra-high-performance liquid chromatography (UHPLC) and mass spectrometry (MS) settings; Figure S1: screening for lipid species with a significant change in relative abundance using volcano plot analysis and heatmap clustering; Figure S2: relative abundance of four selected lipid species; Figure S3: the relative abundance of some lipid classes differs significantly among the cell groups; Figure S4: lipid identification workflow; Figure S5: MS/MS spectra of the precursor ions of representative species of PC; PE; PI; PS and PG.
