# Supplementary material for: A UHPLC-Mass Spectrometry View of Human Melanocytic Cells Uncovers Potential Lipid Biomarkers of Melanoma
Source: Int J Mol Sci. 2021 Nov 8;22(21):12061. doi: 10.3390/ijms222112061 (PMC8585039; doi:10.3390/ijms222112061)
Supplement: Supplementary file 1 [file ijms-22-12061-s001.zip › ijms-1424829-supplementary.pdf]

Supplementary Materials

A UHPLC-MASS Spectrometry View of Human Melanocytic Cells Uncovers Potential Lipid Biomarkers of Melanoma

Arantza Pérez-Valle [1], Beatriz Abad-García [2], Olatz Fresnedo [3], Gabriel Barreda-Gómez [4], Patricia Aspichueta [3,5], Aintzane Asumendi [1,5], Egoitz Astigarraga [4], José A. Fernández [6], María Dolores Boyano [1,5,*], Begoña Ochoa [3,*].

[1] Department of Cell Biology and Histology, Faculty of Medicine and Nursing, University of the Basque Country UPV/EHU, Leioa, Spain; arantza.perezv@ehu.eus (A.P-V.); aintzane.asumendi@ehu.eus (A.A.); lola.boyano@ehu.eus (M.D.B.)
[2] Central Analysis Service, Faculty of Science and Technology, University of the Basque Country UPV/EHU, Leioa, Spain; beatriz.abad@ehu.eus
[3] Department of Physiology, Faculty of Medicine and Nursing, University of the Basque Country UPV/EHU, Leioa, Spain; olatz.fresnedo@ehu.eus (O.F.); patricia.aspichueta@ehu.eus (P.A.); begona.ochoa@ehu.eus (B.O.)
[4] IMG Pharma Biotech S.L., Bizkaia Technological Park, Zamudio, Spain; gabriel.barreda@imgpharma.com (G.B.); egoitz.astigarraga@imgpharma.com (E.A.)
[5] Biocruces-Bizkaia Research Institute, Cruces University Hospital, Barakaldo, Spain
[6] Department of Physical Chemistry, Faculty of Science and Technology, University of the Basque Country UPV/EHU, Leioa, Spain; josea.fernandez@ehu.eus (J.A.F.)

* Correspondence: BO: begona.ochoa@ehu.eus; MDB: lola.boyano@ehu.eus

Table S1. List of lipid species identified by lipidomics in melanocytic cells, along with the relative intensity in each independent sample and the corresponding peak markers (mass to charge ratio (*m*/*z*) and retention time (RT) pairs). Whenever possible, annotation of identified fatty acids was performed at the *sn* position-level. The ether (O) and vinyl ether (P) bonds of alkyls at the *sn*-1 position of the glycerol backbone were also characterized. HeM, human epidermal melanocytes; NM, nevi melanocytes; PM, primary melanoma; MM, metastatic melanoma. PC, phosphatidylcholine; PC(O/P), ether phosphatidylcholine; LPC, lysophosphatidylcholine; SM, sphingomyelin, Cer, ceramide; HexCer, hexosylceramide; CE, cholesteryl ester; TG, triglyceride; DG, diglyceride; PE, phosphatidylethanolamine; PE(O/P), ether phosphatidylethanolamine; LPE, lysophosphatidylethanolamine; PI, phosphatidylinositol; PG, phosphatidylglycerol; PS, phosphatidylserine; FA, fatty acid.

Table S2. Ultra-High Performance Liquid Chromatography (UHPLC) and Mass Spectrometry (MS) settings.

Figure S1. Screening for lipid species with significant change in relative abundance using volcano plot analysis and heatmap clustering. To identify lipids differentially expressed between the groups, volcano plots and heatmap clusters with fold-change > 2 and *p*-value ≤ 0.05

by Student's *t*-test using Benjamini-Hochberg as a multiple testing correction were also performed. Heatmap clustering was visualized for fold-change of potential lipid biomarkers in all ESI+ and ESI- comparisons, showing the level of relative increase (red) and decrease (blue) in peak intensity. In volcano plots, red and blue indicate up- and down-regulated species, respectively. HeM, human epidermal melanocytes; NM, nevus melanocytes; PM, primary melanoma; MM, metastatic melanoma.

Figure S2. Relative abundance of four selected lipid species. The relative abundance of some lipid species increases in neoplastic melanoma, as exemplified by phosphatidylcholine ethers PC O-16:0/18:1 and PC O-16:0/16:0 (**A**), whereas abundance of other lipids decrease markedly, as it occurs with sphingomyelin SM d18:1/16:0 and the phosphatidylethanolamine ether PE P-18:0/22:4 (**B**).

Figure S3. The relative abundance of some lipid classes differ significantly among the cell groups. Relative abundance of the total PC, PC(O), LPC, PE, PE(P), LPE, PI, PG, and PS (**A**), and of the total Cer+, Cer-, SM, HexCer, FA, CE, TG and DG (**B**). The data were estimated as the sum of the intensity values shown in Table S1 and they are expressed as the mean ± SD. Multiple comparisons were performed by one-way ANOVA with a multiple testing correction test. The table in **C** shows the *p*-values for the Levene test to determine variances equivalences used to select the post-hoc method (Tukey if $p \geq 0.05$ and Games-Howell if $p \leq 0.05$), one-way ANOVA *p*-values, and Tukey's or Games-Howell's[1] test *p*-values for the six pairwise comparisons with the minimum significance in the comparison. *$p \leq 0.05$; ** $p \leq 0.01$; *** $p \leq 0.001$; NS, not significant. The numbers highlighted in red and blue denote significant increases and decreases, respectively. HeM, human epidermal melanocytes; NM, nevus melanocytes; PM, primary melanoma; MM, metastatic melanoma.

Figure S4. Lipid identification workflow. (**A**) Fragmentation scheme of PI ions; (**B**) extracted ion chromatogram of [PI(18:1/20:4)-H]- in lipid extracts of human melanocytic cells samples; (**C**) MS/MS spectrum of [PI(18:1/20:4)+NH4]+ in ESI+ and (**D**) of [PI(18:1/20:4)-H]- in ESI- , and (**E**, **F**) comparison of the exact masses of precursor ions and unknown lipid products with those of a database and annotation.

Figure S5. MS/MS spectra of the precursor ions of three representative species of PC, PE, PI, PS and PG. For PC (**A**) and PE (**B**), the intensity of the carboxylate ion peak corresponding to the FA at the *sn*-2 position was always higher than that at *sn*-1. However, the relative intensity of carboxylate product ions displayed in the MS/MS at the *sn*-1 was more intense than that at *sn*-2 fatty acyls for PI (**C**), PS (**D**) and PG (**E**).

Table S1. List of lipid species identified in the positive and negative ion mode along with their corresponding peak markers (mass to charge ratio (*m/z*) and retention time (RT) pairs) and relative abundance in each independent sample.

Note: In this file, decimals are represented by commas.

Lipid class	Lipid species (as adduct)	C in FA	Double bonds in FA		
		No.	No.	Exptl *m/z*	Exptl RT
	Positive ion mode				
PC	[PC(14:0_16:1)+H]+	30	1	704,5209	3,44
	[PC(14:0_16:0)+H]+	30	0	706,5414	3,98
	[PC(17:0/14:1)+H]+	31	1	718,5396	3,79
	[PC(16:1/16:1)+H]+	32	2	730,5407	3,60
	[PC(16:0/16:1)+H]+	32	1	732,5547	4,08
	[PC(16:0/16:0)+H]+	32	0	734,5680	4,60
	[PC(16:0/18:2)+H]+	34	2	758,5685	4,20
	[PC(16:0/18:1)+H]+	34	1	760,5875	4,66
	[PC(16:0/18:0)+H]+	34	0	762,6008	5,26
	[PC(35:1)+H]+	35	1	774,6017	4,95
	[PC(16:0/20:4)+H]+	36	4	782,5697	4,05
	[PC(16:0/20:3)+H]+	36	3	784,5831	4,32
	[PC(36:2)+H]+	36	2	786,6052	4,74
	[PC(18:0/18:1)+H]+	36	1	788,6176	5,27
	[PC(16:0/22:5)+H]	38	5	808,5850	4,21
	[PC(38:3)+H]+	38	3	812,6182	4,92
	[PC(18:0/20:2)+H]+	38	2	814,6337	5,29
	[PC(38:1)+H]+	38	1	816,6492	5,81
	[PC(40:6)+H]+	40	6	834,6019	4,30
	[PC(40:5)+H]+	40	5	836,6148	4,69
	[PC(40:4)+H]+	40	4	838,6320	5,06
	[PC(40:2)+H]+	40	2	842,6666	5,81
	Total PC - phosphatidylcholine -				
PC(O/P)	[PC(O-30:0)+H]+	30	0	692,5601	4,39
	[PC(O-32:1)+H]+	32	1	718,5743	4,48
	[PC(O-16:0/16:0)+H]+	32	0	720,5921	5,02
	[PC(O-16:0/18:1)+H]+	34	1	746,6087	5,08
	[PC(O-36:2)+H]+	36	2	772,6240	5,13
	Total PC(O/P) - ether phosphatidylcholine -				
LPC	[LPC(18:1)+H]+	18	1	522,3563	1,17
	Total LPC - lysophosphatidylcholine -				
SM	[SM(d18:1/14:0)+H]+	32	1	675,5428	3,27
	[SM(d18:0/14:0)+H]+	32	0	677,5562	3,38
	[SM(d18:1/15:0)+H]+	33	1	689,5615	3,60
	[SM(d34:2)+H]+	34	2	701,5585	3,39
	[SM(d18:1/16:0)+H]+	34	1	703,5790	3,93
	[SM(d18:0/16:0)+H]+	34	0	705,5837	3,97
	[SM(d18:2/18:1)+H]+	36	3	727,5742	2,03
	[SM(d18:1/18:0)+H]+	36	1	731,6078	4,57
	[SM(d18:0/20:0)+H]+	38	0	761,6558	5,33
	[SM(d18:1/22:1)+H]+	40	2	785,6613	5,23
	[SM(d18:1/22:0)+H]+	40	1	787,6701	5,76

	[SM(41:2)+H]+	41	2	799,6661	5,48
	[SM(d18:1/23:0)+H]+	41	1	801,6830	6,05
	[SM(d18:2/24:1)+H]+	42	3	811,6437	5,29
	[SM(d18:1/24:1)+H]+	42	2	813,6875	5,74
	[SM(d18:1/24:0)+H]+	42	1	815,7000	6,31
	[SM(d18:1/26:1)+H]+	44	2	841,7153	6,27
	Total SM - sphingomyelin -				
Cer (+)	[Cer(d18:1/24:1)+Na]+	42	2	670,6082	6,50
	[Cer(d18:1/24:0)+Na]+	42	1	672,6248	6,97
	Total Cer (+) - ceramide -				
HexCer	[HexCer(d18:1/22:0)+Na]+	40	1	806,6603	5,91
	[HexCer(d18:1/24:1)+Na]+	42	2	832,6644	5,89
	[HexCer(d18:1/24:0)+Na]+	42	1	834,6785	6,39
	Total HexCer -hexosylceramide -				
CE	[CE(14:1)+NH4]+	14	1	612,5709	5,92
	[CE(15:0)+NH4]+	15	0	628,5992	6,12
	[CE(16:2)+NH4]+	16	2	638,5806	5,99
	[CE(17:1)+NH4]+	17	1	654,6161	6,59
	[CE(18:1)+NH4]+	18	1	668,6347	9,14
	[CE(20:4)+NH4]+	20	4	690,6231	5,80
	Total CE -cholesteryl ester -				
TG	[TG(42:0)+NH4]+	42	0	740,6774	7,77
	[TG(44:1)+NH4]+	44	1	766,6961	7,81
	[TG(44:0)+NH4]+	44	0	768,7065	8,08
	[TG(46:2)+NH4]+	46	2	792,7099	7,86
	[TG(46:1)+NH4]+	46	1	794,7218	8,11
	[TG(46:0)+NH4]+	46	0	796,7408	8,37
	[TG(47:1)+NH4]+	47	1	808,7387	8,24
	[TG(47:0)+NH4]+	47	0	810,7530	8,48
	[TG(48:3)+NH4]+	48	3	818,7232	7,91
	[TG(48:2)+NH4]+	48	2	820,7400	8,15
	[TG(48:1)+NH4]+	48	1	822,7576	8,39
	[TG(48:0)+NH4]+	48	0	824,7668	8,60
	[TG(49:2)+NH4]+	49	2	834,7553	8,30
	[TG(49:1)+NH4]+	49	1	836,7702	8,51
	[TG(49:0)+NH4]+	49	0	838,7841	8,71
	[TG(50:4)+NH4]+	50	4	844,7390	7,99
	[TG(50:3)+NH4]+	50	3	846,7561	8,19
	[TG(50:2)+NH4]+	50	2	848,7715	8,42
	[TG(50:1)+NH4]+	50	1	850,7849	8,65
	[TG(50:0)+NH4]+	50	0	852,7977	8,80
	[TG(51:3)+NH4]+	51	3	860,7723	8,33
	[TG(51:2)+NH4]+	51	2	862,7866	8,53
	[TG(51:1)+NH4]+	51	1	864,7989	8,74
	[TG(52:6)+NH4]+	52	6	868,7380	
	[TG(52:5)+NH4]+	52	5	870,7568	7,99
	[TG(52:4)+NH4]+	52	4	872,7720	8,22
	[TG(52:3)+NH4]+	52	3	874,7882	8,44
	[TG(52:2)+NH4]+	52	2	876,8059	8,66
	[TG(52:1)+NH4]+	52	1	878,8138	8,84
	[TG(52:0)+NH4]+	52	0	880,8299	9,05
	[TG(53:3)+NH4]+	53	3	888,7983	8,53

	[TG(53:2)+NH4]+	53	2	890,8117	8,73
	[TG(54:6)+NH4]+	54	6	896,7690	8,02
	[TG(54:5)+NH4]+	54	5	898,7851	8,24
	[TG(54:4)+NH4]+	54	4	900,8043	8,47
	[TG(54:3)+NH4]+	54	3	902,8141	8,68
	[TG(54:2)+NH4]+	54	2	904,8321	8,86
	[TG(54:1)+NH4]+	54	1	906,8446	9,05
	[TG(56:6)+NH4]+	56	6	924,8008	8,38
	[TG(56:5)+NH4]+	56	5	926,8162	8,55
	[TG(56:4)+NH4]+	56	4	928,8324	8,73
	[TG(56:3)+NH4]+	56	3	930,8490	8,89
	[TG(56:2)+NH4]+	56	2	932,8628	9,09
	[TG(56:1)+NH4]+	56	1	934,8772	9,26
	[TG(58:6)+NH4]+	58	6	952,8308	8,58
	[TG(58:5)+NH4]+	58	5	954,8464	8,77
	[TG(58:4)+NH4]+	58	4	956,8638	8,92
	[TG(58:3)+NH4]+	58	3	958,8792	9,12
	[TG(58:2)+NH4]+	58	2	960,8942	9,32
	[TG(58:1)+NH4]+	58	1	962,9088	9,50
	[TG(60:2)+NH4]+	60	2	988,9244	9,51
	Total TG - triglyceride -				
DG	[DG(32:1)+Na]+	32	1	589,5244	5,41
	[DG(32:0)+Na]+	32	0	591,5103	5,90
	[DG(34:1)+Na]+	34	1	617,5137	5,95
	[DG(34:0)+Na]+	34	0	619,5272	6,42
	[DG(36:2)+Na]+	36	2	643,5308	6,02
	[DG(36:1)+Na]+	36	1	645,5461	6,46
	[DG(36:0)+Na]+	36	0	647,4704	6,80
	Total DG -diglyceride -				
	Negative ion mode				
PE	[PE(16:0/16:1)-H]-	32	1	688,4999	4,25
	[PE(34:2)-H]-	34	2	714,5048	4,32
	[PE(34:1)-H]-	34	1	716,5255	4,85
	[PE(16:0/20:4)-H]-	36	4	738,5086	4,28
	[PE(18:1/18:2)-H]-	36	3	740,5206	4,49
	[PE(18:0/18:1)-H]-	36	1	744,5509	5,43
	[PE(18:1/20:4)-H]-	38	5	764,5331	4,36
	[PE(18:1/20:3)-H]-	38	4	766,5422	4,90
	[PE(18:0/20:3)-H]-	38	3	768,5539	5,25
	[PE(18:0/20:2)-H]-	38	2	770,5688	5,48
	[PE(18:1/22:4)-H]-	40	5	792,5683	4,66
	[PE(18:0/22:4)-H]-	40	4	794,5681	5,25
	Total PE - phosphatidylethanolamine -				
PE(O/P)	[PE(P-16:0/16:1)-H]-	32	1	672,4963	4,59
	[PE(P-16:0/16:0)-H]-	32	0	674,5109	5,12
	[PE(P-16:0/18:1)-H]-	34	1	700,5258	5,17
	[PE(P-18:0/17:2)-H]-	35	2	712,5252	4,98
	[PE(P-16:0/20:5)-H]-	36	5	720,4969	4,20
	[PE(P-16:0/20:4)-H]-	36	4	722,5110	4,60
	[PE(P-18:0/18:2)-H]-	36	2	726,5413	5,23
	[PE(P-18:0/18:1)-H]-	36	1	728,5565	5,74
	[PE(P-16:0/22:6)-H]-	38	6	746,5122	4,42

	[PE(P-38:5)-H]-	38	5	748,5288	4,65
	[PE(P-38:4)-H]-	38	4	750,5421	5,17
	[PE(P-38:3)-H]-	38	3	752,5569	5,45
	[PE(P-38:2)-H]-	38	2	754,5736	5,76
	[PE(P-38:1)-H]-	38	1	756,5895	6,23
	[PE(P-18:1/22:6)-H]-	40	7	772,5285	4,50
	[PE(P-18:0/22:6)-H]-	40	6	774,5456	5,03
	[PE(P-40:5)-H]-	40	5	776,5580	5,18
	[PE(P-18:0-22:4)-H]-	40	4	778,5733	5,56
	Total PE(O/P) -ether phosphatidylethanolamine -				
LPE	[LPE(16:0)-H]-	16	0	452,2763	1,10
	[LPE(18:1)-H]-	18	1	478,2904	0,58
	[LPE(18:0)-H]-	18	0	480,3087	1,48
	[LPE(20:4)-H]-	20	4	500,2766	0,85
	Total LPE -lysophosphatidylethanolamine -				
PI	[PI(32:1)-H]-	32	1	807,5026	3,10
	[PI(16:1/18:1)-H]-	34	2	833,5197	3,18
	[PI(16:1/18:0)-H]-	34	1	835,5326	3,67
	[PI(16:0/20:4)-H]-	36	4	857,5176	3,14
	[PI(36:3)-H]-	36	3	859,5326	3,39
	[PI(18:0/18:2)-H]-	36	2	861,5506	3,75
	[PI(16:0/20:1)-H]-	36	1	863,5633	4,26
	[PI(18:1/20:4)-H]-	38	5	883,5350	3,24
	[PI(18:0/20:4)-H]-	38	4	885,5518	3,75
	[PI(18:0/20:3)-H]-	38	3	887,5620	3,93
	[PI(18:0/20:2)-H]-	38	2	889,5792	4,31
	[PI(40:6)-H]-	40	6	909,5487	3,59
	[PI(40:5)-H]-	40	5	911,5645	3,76
	[PI(40:4)-H]-	40	4	913,5779	4,14
	[PI(40:3)-H]-	40	3	915,5961	4,24
	Total PI -phosphatidylinositol -				
PG	[PG(32:0)-H]-	32	0	721,4996	4,21
	[PG(18:0/16:1)-H]-	34	1	747,5139	4,47
	[PG(18:0_16:0)-H]-	34	0	749,5321	4,66
	[PG(18:1/18:2)-H]-	36	3	771,5170	3,13
	[PG(18:1/18:1)-H]-	36	2	773,5329	4,51
	[PG(36:1)-H]-	36	1	775,5451	5,04
	[PG(18:1/20:2)-H]-	38	3	799,5449	3,62
	[PG(18:1/22:6)-H]-	40	7	819,5173	2,88
	Total PG -phosphatidylglycerol -				
PS	[PS(16:0/18:1)-H]-	34	1	760,5117	3,88
	[PS(36:2)-H]-	36	2	786,5266	3,94
	[PS(36:1)-H]-	36	1	788,5411	4,44
	[PS(18:0/20:4)-H]-	38	4	810,5261	4,95
	[PS(38:2)-H]-	38	2	814,5586	4,50
	[PS(38:1)-H]-	38	1	816,5735	4,25
	[PS(18:0/22:2)-H]-	40	2	842,5904	4,37
	[PS(18:0/22:1)-H]-	40	1	844,6074	4,80
	Total PS -phosphatidylserine -				
Cer	[Cer(d18:1/16:0)-H]-	34	1	536,5073	4,86
	[Cer(d18:1/18:1)-H]-	36	2	562,5230	5,00

	[Cer(d18:1/18:0)-H]-	36	1	564,5353	5,46
	[Cer(d18:2/22:0)-H]-	40	2	618,5809	6,05
	[Cer(d18:1/22:0)-H]-	40	1	620,5964	6,52
	[Cer(d18:1/24:0)-H]-	42	1	648,6301	6,97
	Total Cer (-) - ceramide -				
FA	[FA(14:0-H]-	14	0	227,2029	1,13
	[FA(15:0)-H]-	15	0	241,2150	1,31
	[FA(16:1)-H]-	16	1	253,2172	1,23
	[FA(16:0)-H]-	16	0	255,2331	1,57
	[FA(17:1)-H]-	17	1	267,2318	1,45
	[FA(17:0)-H]-	17	0	269,2466	1,82
	[FA(18:2)-H]-	18	2	279,2350	1,33
	[FA(18:1)-H]-	18	1	281,2496	1,67
	[FA(18:0)-H]-	18	0	283,2635	2,17
	[FA(19:1)-H]-	19	1	295,2640	1,96
	[FA(19:0)-H]-	19	0	297,2789	2,49
	[FA(20:4)-H]-	20	4	303,2335	1,24
	[FA(20:3)-H]-	20	3	305,2482	1,52
	[FA(20:2)-H]-	20	2	307,2624	1,82
	[FA(20:1)-H]-	20	1	309,2815	2,26
	[FA(20:0)-H]-	20	0	311,2934	2,90
	[FA(21:0)-H]-	21	0	325,3097	3,25
	[FA(22:6)-H]-	22	6	327,2320	1,12
	[FA(22:5)-H]-	22	5	329,2466	1,32
	[FA(22:4)-H]-	22	4	331,2637	1,62
	[FA(22:3)-H]-	22	3	333,2795	2,01
	[FA(22:2)-H]-	22	2	335,2944	2,43
	[FA(22:1)-H]-	22	1	337,3101	2,96
	[FA(22:0)-H]-	22	0	339,3245	3,69
	Total FA -fatty acid-				

HeM	HeM	HeM	HeM	Skin melanocytes			NM	NM
**HeMn-LP**	**HeMn-DP**	**HeMn-DP**	**HeMn-MP**	**Mean**	**% Class**		**N6 (P7)**	**N1 (P4)**
34563	36762	61770	59356	48113	0,7		25393	9789
305109	341630	295363	440589	345673	5,0		151646	42985
47165	47168	46947	65099	51595	0,8		44143	39034
34789	82254	134078	123857	93745	1,4		37698	19857
670093	935657	934266	1183635	930912	13,6		443362	175176
286199	460069	401851	615088	440802	6,4		273559	90810
536326	782656	1042423	1305489	916724	13,4		342327	156796
1879978	1929801	1702190	2100044	1903003	27,8		1413131	1093357
17228	34439	26253	41829	29937	0,4		31566	21946
19334	11894	9464	20333	15256	0,2		15958	23042
27818	36045	32486	45732	35520	0,5		33528	36062
199282	202201	222596	374743	249705	3,6		79753	39395
1139260	1352591	1092167	1647948	1307992	19,1		477243	341178
223323	416381	256494	420405	329151	4,8		290626	707077
41880	43331	30567	64683	45115	0,7		39283	45820
24642	56974	53003	79440	53515	0,8		46320	40798
31307	32372	18650	33424	28938	0,4		19379	11672
2372	6207	1945	14172	6174	0,1		3061	2302
5942	6967	3529	7157	5899	0,1		6271	4864
5537	7346	6066	8908	6964	0,1		7004	7301
2739	11737	11218	13352	9762	0,1		6409	5162
2286	2262	2061	4891	2875	0,0		2038	1217
5537174	6836743	6385387	8670175	6857370	100,0		3789699	2915639
10828	7222	6854	9236	8535	4,1		3369	1036
33123	19046	31736	34154	29515	14,0		5964	3244
22739	33959	27810	39840	31087	14,8		6625	2547
97733	138525	96141	151702	121025	57,5		17501	13630
18008	20248	16042	26360	20165	9,6		3400	2275
182431	219001	178583	261292	210327	100,0		36859	22731
1	2828	843	743	1232			554	751
				1232	100,0			
20439	9708	17885	20391	17106	2,2		9869	6056
3653	1654	2832	5606	3436	0,4		1324	1012
2233	1621	1457	2866	2044	0,3		3380	3083
3724	3672	5126	10740	5815	0,7		9402	8536
382108	295780	331967	433695	360888	46,4		267529	180613
43560	31970	38573	98447	53138	6,8		21649	17402
7149	6116	8036	6815	7029	0,9		5563	6179
39975	59504	49724	34992	46049	5,9		7553	10406
873	1615	1207	2247	1486	0,2		401	643
13494	12755	16664	21911	16206	2,1		7633	8397
11154	11413	11064	14061	11923	1,5		18930	19146

6917	3988	4979	7574	5865	0,8	6198	6792
1157	1632	946	1048	1196	0,2	2751	2763
15290	18318	24618	49291	26880	3,5	27521	19532
188297	209023	180801	247092	206303	26,5	146533	92827
9647	9402	7108	11053	9303	1,2	44458	27638
2082	2699	2976	2946	2676	0,3	3336	1151
751753	680869	705964	970774	777340	100,0	584030	412176
7321	11019	10854	9663	9714	84,9	5850	8076
1430	1855	1836	1766	1722	15,1	4220	5364
8751	12874	12690	11429	11436	100,0	10070	13440
8083	7094	8448	7549	7793	13,1	1911	5909
33198	44204	37555	37049	38002	63,9	7487	14657
10623	16268	13335	14511	13684	23,0	9271	14019
51904	67566	59338	59109	59479	100,0	18669	34586
1180	2810	1268	1469	1682	14,2	298	470
2841	3297	3320	5309	3692	31,2	2445	4364
1273	4657	1496	2197	2406	20,3	397	400
606	639	610	593	612	5,2	593	836
314	261	302	674	388	3,3	280	396
4125	1404	4876	1880	3071	25,9	2302	2086
10338	13068	11872	12122	11850	100,0	6315	8553
2383	2828	1440	1919	2142	0,1	1076	1233
3264	11183	5216	2539	5550	0,2	948	1951
6195	11218	4689	3765	6467	0,2	2137	2394
4627	31725	13923	5756	14008	0,4	1582	3671
15660	92330	29356	15129	38119	1,1	3390	7614
12481	35743	13605	9911	17935	0,5	4838	5825
6059	6980	5726	4791	5889	0,2	2330	3985
8450	5709	5393	5508	6265	0,2	3949	4237
3627	37457	21497	5614	17049	0,5	1541	3827
27317	263121	98119	27695	104063	2,9	5279	11165
54306	255213	79796	41589	107726	3,0	9633	16723
21570	54310	24613	19882	30094	0,8	11931	13640
4999	9713	6100	4446	6315	0,2	1998	3885
8571	11438	7685	6953	8662	0,2	3075	5801
5713	4770	3876	3534	4473	0,1	2579	3069
3171	29532	18536	5143	14096	0,4	1601	4242
27097	303740	182197	47749	140196	3,9	9916	18416
159470	822939	416570	188537	396879	11,1	39228	68782
83916	334303	142012	101940	165543	4,6	30894	47681
17581	48538	22886	22350	27839	0,8	13532	15123
3475	7755	4619	3253	4776	0,1	1449	2760
8770	16790	8506	6604	10167	0,3	2663	5773
7003	10967	6438	6236	7661	0,2	2518	4398
5205	7190	6935	4933	6066	0,2	4630	4597
17132	37645	33344	19647	26942	0,8	14849	16974
42134	216428	164689	65014	122066	3,4	29128	38767
166244	1053686	631243	293917	536272	15,0	53631	87357
286967	989372	490194	326595	523282	14,6	62091	122659
49635	240748	84519	61957	109215	3,0	19433	32862
6236	22068	10130	9469	11976	0,3	7140	7716
5932	22407	9711	10280	12083	0,3	2885	6214

10457	22048	13482	11600	14397	0,4		4739	8445
12890	23861	21251	14072	18018	0,5		10517	12216
22088	69720	59375	28844	45007	1,3		14405	18337
55676	329656	220616	116517	180617	5,0		27067	35749
206410	713935	468183	396833	446340	12,5		54037	105583
64089	309012	122442	116873	153104	4,3		20576	42999
8948	40470	13708	22948	21518	0,6		4730	8315
7559	22432	13553	7638	12795	0,4		1510	4249
8582	77533	44729	18704	37387	1,0		2643	5875
8371	115937	52089	27437	50958	1,4		4006	7675
12634	102276	38895	31303	46277	1,3		3537	6954
5666	32405	17665	11164	16725	0,5		2512	4082
2041	5646	2444	2549	3170	0,1		1085	1580
3233	11540	6556	4402	6433	0,2		742	1297
2229	30770	13348	7522	13467	0,4		1073	1889
1569	20522	8644	5491	9056	0,3		862	1429
2549	19091	7397	5284	8580	0,2		946	1818
1987	9203	5822	3159	5043	0,1		1183	1525
1160	4965	1209	1316	2162	0,1		993	958
854	4851	1500	1546	2188	0,1		663	948
1514185	6963720	3686470	2167857	3583058	100,0		509698	845265
10066	18592	15831	14464	14738	9,1		16834	4307
5105	8057	9427	8212	7700	4,8		9068	5691
14333	28969	15262	19635	19549	12,1		5841	4509
8722	11662	8528	11967	10220	6,3		13108	16076
17704	38908	19901	27236	25937	16,0		5161	4157
4438	7644	3571	6712	5591	3,5		3368	3344
47573	87817	84698	93078	78292	48,3		53649	82194
107941	201648	157219	181303	162028	100,0		107029	120277
15133	16023	16947	19426	16882	4,9		7020	3165
40939	39590	69504	62200	53058	15,3		10959	3685
35678	41402	47931	48971	43495	12,6		29155	12315
2105	3816	4449	5418	3947	1,1		4847	1854
13750	15087	21044	21682	17891	5,2		5662	1979
63591	96243	77838	103756	85357	24,7		45492	37317
21924	24323	24239	23439	23481	6,8		18288	10613
12886	21254	23362	28026	21382	6,2		21445	19617
10293	45330	46085	48841	37637	10,9		20348	9664
15179	20800	16641	22145	18691	5,4		9428	3701
9942	13274	12660	17135	13253	3,8		8502	7518
7240	13495	11651	12259	11161	3,2		3695	2372
248662	350636	372350	413297	346236	100,0		184843	113799
7678	2043	7016	6304	5760	1,7		2615	752
3060	1503	2751	4142	2864	0,8		1385	719
85594	28575	76865	81533	68142	19,9		22354	15663
1433	360	721	776	823	0,2		584	501
1631	523	1242	1659	1264	0,4		1650	880
18795	9475	25634	33037	21735	6,3		21422	13432
79329	37439	50979	51852	54900	16,0		14798	8403
17273	5073	15108	13785	12810	3,7		10889	7885
7046	3339	7553	11897	7459	2,2		5669	3280

33743	19058	44566	71224	42148	12,3	15113	12010
16826	20679	54245	52048	35949	10,5	32050	28319
5518	16084	52310	32536	26612	7,8	12754	7687
8469	5911	20522	13264	12041	3,5	3549	1905
3706	1532	5194	5034	3866	1,1	1322	785
9510	2713	9839	11746	8452	2,5	3646	2278
3551	1889	3854	7728	4255	1,2	4555	3780
10158	5677	15576	21603	13254	3,9	4760	3168
3855	12265	37680	28249	20512	6,0	4455	2981
317173	174140	431654	448418	342846	100,0	163572	114426
1208	9252	348	429	2809	13,8	782	444
2064	16579	622	696	4990	24,4	1084	979
1944	4485	1785	2148	2591	12,7	2818	3223
2096	36309	651	1049	10026	49,1	1527	882
7313	66625	3406	4323	20417	100,0	6211	5529
1817	2438	2075	2495	2206	0,9	1768	1762
13246	22411	18458	25825	19985	8,4	5911	4815
10756	10288	7020	13445	10377	4,3	10496	10243
2136	2649	2638	2624	2512	1,1	2076	1929
7426	8606	9413	9434	8720	3,6	3303	2308
38213	34821	26416	39827	34819	14,6	11707	8325
21450	15841	13281	29959	20133	8,4	10730	9967
20407	18752	11957	19723	17710	7,4	8116	6404
43651	54877	43468	59556	50388	21,1	27816	25846
14616	49591	60307	51865	44095	18,4	17240	10017
9301	17149	20202	21109	16940	7,1	5598	2159
1410	1306	1016	1813	1387	0,6	1668	1221
2540	2253	2076	3376	2561	1,1	1801	1407
2094	2885	2387	2819	2546	1,1	1438	1023
2681	5445	5168	5768	4766	2,0	2964	2558
191745	249311	225883	289638	239144	100,0	112633	89983
913	363	578	896	688	1,7	896	469
2810	1046	2209	3634	2425	6,1	2152	1231
15181	8824	20406	31611	19005	47,5	7347	5800
4719	5280	4528	5221	4937	12,3	1457	1375
4519	1430	4873	5877	4175	10,4	1710	1257
1957	922	1997	3510	2097	5,2	2314	1471
2643	6117	5342	6334	5109	12,8	1621	1462
1280	1361	1623	1918	1546	3,9	2522	3610
34021	25342	41556	59002	39980	100,0	20018	16675
6098	14681	12708	12497	11496	5,9	11412	4094
25374	23658	22732	25149	24228	12,4	8213	3050
90535	135809	95020	105009	106593	54,6	81904	61215
5579	5079	5252	4766	5169	2,6	4002	2706
7708	10738	8821	11188	9614	4,9	5716	2263
12646	14395	15969	16909	14980	7,7	10447	8279
3844	3590	4392	4302	4032	2,1	2686	1945
19921	18315	18265	20538	19260	9,9	13214	10885
171705	226265	183160	200358	195372	100,0	137593	94437
67247	53381	82144	86542	72329	67,6	18029	33491
3322	5065	5405	5431	4806	4,5	2296	1407

11288	18400	21763	13395	16212	15,2		2570	4507
3686	3882	4044	4932	4136	3,9		1476	2757
2595	2998	2983	3002	2895	2,7		2788	6730
5566	6351	4711	9813	6610	6,2		14207	18834
93704	90079	121049	123116	106987	100,0		41367	67726
64427	62118	45049	43629	53806	1,0		79602	66050
78288	27368	29036	24171	39715	0,7		85873	45074
110395	211297	152397	141866	153989	2,8		49943	58944
1403846	1204387	1493732	819599	1230391	22,7		1646872	1679505
26929	11782	14607	10491	15952	0,3		14800	17612
117467	37203	45565	46058	61573	1,1		77497	85995
68364	130332	114097	87404	100049	1,8		38332	43625
1264280	1930495	985268	1595528	1443893	26,7		376580	436375
1259774	1916801	3037342	1257738	1867914	34,5		2170107	2565467
10190	6136	3438	7528	6823	0,1		3199	6460
41459	16115	17033	12144	21688	0,4		20713	25580
94033	37821	16755	32704	45328	0,8		43103	77525
19848	185296	104052	102401	102899	1,9		24246	19115
16412	75799	39987	59589	47947	0,9		10481	7307
39258	76819	30039	63745	52465	1,0		11337	9712
60130	41201	62010	30787	48532	0,9		48866	77219
22396	8415	7427	6308	11136	0,2		9537	21510
5296	3861	1568	6019	4186	0,1		3228	4601
18358	6039	3462	9223	9271	0,2		4930	4673
20720	13095	7404	15174	14098	0,3		5282	6309
2777	41264	15277	20281	19900	0,4		5530	3751
4611	12956	6305	9503	8344	0,2		2384	1493
8588	13489	6340	11590	10002	0,2		2977	3147
71473	42998	35876	22185	43133	0,8		32163	91688
4829319	6113088	6274065	4435662	5413034	100,0		4767581	5358736

NM	NM	NM	NM	NM	NM	Nevi melanocytes	
N6 (P9)	N4 (P5)	N3 (P4)	N3 (P6)	N2 (P4)	N2 (P5)	Mean	% Class
24525	13070	13579	32583	59075	129205	38402	0,8
151684	85524	106024	233423	143126	303463	152234	3,2
55792	36103	54872	67000	47967	56174	50136	1,0
42840	19811	33138	77133	106467	274444	76423	1,6
407343	246571	315981	648656	682135	1185228	513056	10,7
291857	116827	290975	432850	159685	343943	250063	5,2
394653	294149	252874	549398	743141	1442250	521949	10,9
1412189	1191374	1381686	2019000	1317606	2161282	1498703	31,3
38768	9168	48469	75891	20373	54197	37547	0,8
35045	6365	25615	52039	6551	10947	21945	0,5
39074	16999	72818	94395	36732	76533	50768	1,1
120857	91036	66306	121674	196418	440135	144447	3,0
750100	970939	602288	950477	558988	1164937	727019	15,2
480411	131405	693059	1060245	165529	426754	494388	10,3
57835	15346	145824	183649	24311	67015	72386	1,5
67919	31878	47674	89722	68156	202560	74378	1,6
23673	15945	12087	25989	12806	27599	18644	0,4
4002	1818	2593	4889	2110	4944	3215	0,1
6734	3215	12291	11702	3743	6736	6945	0,1
10148	4694	17900	16318	9931	17972	11408	0,2
10182	4919	13582	18398	14856	54420	15991	0,3
2116	2320	1296	3310	2273	3409	2247	0,0
4427746	3309477	4210932	6768744	4381979	8454147	4782295	100,0
2339	4612	1243	2022	3659	3292	2697	5,3
6481	15477	3976	6063	16778	12982	8871	17,4
5344	11618	3349	6609	9687	11285	7133	14,0
25139	66370	19103	30278	22212	26529	27595	54,0
5163	13269	2663	1267	4332	5997	4796	9,4
44466	111346	30334	46238	56667	60086	51091	100,0
769	464	778	610	487	612	628	
						628	100,0
8544	7853	6464	12453	11639	22553	10679	1,7
1239	1545	1030	1418	1345	2937	1481	0,2
4330	937	2996	5281	1417	2824	3031	0,5
9385	2309	5861	12055	9761	15623	9116	1,4
274863	213261	212016	372762	238857	518075	284747	44,3
22770	31552	24660	32137	23001	39907	26635	4,1
6424	6603	5720	6325	5465	5189	5933	0,9
7742	26483	19400	13372	5823	8649	12428	1,9
486	797	759	842	553	711	649	0,1
8641	9030	5496	7693	8604	15139	8829	1,4
17677	9415	11546	16337	28181	45292	20816	3,2

9603	4195	5889	9549	4603	8409	6905	1,1
2677	831	2155	2570	1183	1788	2090	0,3
33012	13557	16339	32045	27763	59735	28688	4,5
179581	201594	102034	218942	111202	369199	177739	27,7
32463	13995	23129	29996	45523	93531	38842	6,0
2219	5279	1431	1841	5514	7812	3573	0,6
621655	549233	446924	775620	530435	1217372	642181	100,0
12860	8389	7303	8881	6570	12409	8792	65,4
7047	1894	3135	3534	4679	7320	4649	34,6
19907	10284	10437	12415	11249	19729	13441	100,0
2317	6650	4764	4384	1728	1866	3691	14,2
10772	25198	20274	15394	4440	6005	13028	50,0
8242	9498	11460	10636	5267	6186	9322	35,8
21331	41346	36497	30414	11436	14057	26042	100,0
432	1266	621	563	296	570	565	6,7
5131	3479	3286	3767	2383	5034	3736	44,4
421	3941	646	757	417	896	984	11,7
720	968	527	873	473	725	714	8,5
326	352	378	251	255	375	327	3,9
2055	6270	821	1329	989	817	2084	24,8
9085	16276	6279	7540	4814	8416	8409	100,0
1161	6414	1424	1598	930	1059	1862	0,1
1624	5664	2727	2802	1855	3485	2632	0,2
2491	14342	3342	3386	2731	3238	4258	0,3
2501	6626	5780	5176	4370	11058	5095	0,4
5526	20320	12583	11555	9616	23653	11782	0,9
5282	27337	8902	8643	6732	11388	9868	0,7
3800	12353	6107	6658	3113	2570	5115	0,4
4109	26507	5525	6290	3477	2417	7064	0,5
2183	4015	6416	4650	5473	19387	5937	0,4
7931	21756	18806	16565	18107	66803	20802	1,5
12130	40986	30037	25329	23774	71394	28751	2,1
13363	34954	18706	18736	15937	23153	18803	1,4
3199	7270	6194	6795	2743	3821	4488	0,3
4795	15302	8809	9195	4065	4665	6963	0,5
3080	14952	4162	4771	2312	2247	4647	0,3
2296	3380	7861	5478	6770	32323	7994	0,6
13498	24984	30860	25480	33919	136516	36699	2,7
50279	115425	123390	99606	107698	354946	119919	8,7
40611	73816	80776	63432	58965	142380	67320	4,9
15076	23313	21220	19572	18717	26718	19159	1,4
2147	2805	4487	4194	1910	5190	3118	0,2
4296	6673	9104	8648	3845	8749	6219	0,5
3903	7611	6499	5920	3336	5168	4919	0,4
5212	5483	5935	5148	4778	8363	5518	0,4
18808	18580	20084	19964	19264	47914	22055	1,6
39718	43299	53421	52457	53709	177101	60950	4,4
68483	158338	151262	127695	129337	469353	155682	11,3
84353	246618	246652	180004	118482	397951	182351	13,2
26295	50911	66958	46782	29930	81573	44343	3,2
7320	8829	11749	10419	8243	11503	9115	0,7
4203	4255	8201	7898	4216	9867	5967	0,4

5832	7350	13175	11270	7291	12093	8774	0,6
12485	12766	14653	14157	14290	39331	16302	1,2
18475	19735	24697	22816	28779	120357	33450	2,4
32346	58349	54532	50986	65364	293731	77265	5,6
71185	245985	204066	166214	112197	369540	166101	12,0
26355	68272	89817	68036	38440	133861	61044	4,4
6046	10373	15295	12053	6366	31258	11804	0,9
2047	3936	8294	5173	6983	50305	10312	0,7
3430	8704	13210	9885	14621	134668	24130	1,7
5339	16172	16060	14531	16568	128486	26105	1,9
4880	18849	14808	13147	9809	49306	15161	1,1
3257	7594	7353	7150	4431	17171	6694	0,5
1342	2656	2783	2350	1751	2996	2068	0,1
958	1747	3724	2413	4694	39301	6860	0,5
1586	3528	4483	3743	7349	72655	12038	0,9
1171	3159	3138	3239	4103	28486	5698	0,4
1266	4223	3622	3354	2682	11062	3622	0,3
1232	2964	2736	2415	1873	4806	2342	0,2
1028	2057	1526	1378	1065	1789	1349	0,1
760	1509	1641	1626	1325	2302	1347	0,1
660697	1553048	1487591	1230782	1058331	3709457	1381859	100,0
20171	27227	6552	4335	4267	5593	11161	9,3
8569	10785	5602	4989	7221	4991	7115	5,9
5822	15860	8601	7292	6872	12624	8428	7,0
10697	7744	13882	12481	17651	11534	12896	10,8
5489	22400	8619	9294	6770	13112	9375	7,8
3571	4577	5303	6159	3412	4924	4332	3,6
64901	63219	59505	53767	80762	73451	66431	55,5
119221	151812	108066	98318	126955	126229	119738	100,0
6099	5871	3862	5966	17288	18764	8504	4,2
9039	18038	4085	7517	27277	20537	12642	6,2
24943	24492	13242	22644	40767	40396	25994	12,7
4000	1412	2969	6343	4212	4421	3757	1,8
5638	10142	2575	4474	16849	11978	7412	3,6
51076	49755	49059	72179	55746	55873	52062	25,5
17784	13263	15930	21328	16436	16737	16297	8,0
23133	7896	35361	49637	19960	22784	24979	12,2
24008	19865	14139	23435	35704	49996	24645	12,1
10315	11329	5117	9494	11079	14646	9388	4,6
10914	6226	14221	15249	9909	15699	11030	5,4
4852	5185	6628	7621	10441	19596	7549	3,7
191801	173474	167186	245886	265669	291428	204261	100,0
2052	5986	662	1199	3600	3885	2594	1,1
1275	3133	508	845	2163	2462	1561	0,7
27423	80190	12738	21808	31053	45906	32142	14,2
1099	685	430	776	393	484	619	0,3
1729	726	1418	2013	643	1012	1259	0,6
23669	14881	24841	36125	12919	18288	20697	9,1
21120	62158	8830	15834	13879	22482	20938	9,2
13064	15756	7141	11920	9586	15848	11511	5,1
5557	5147	8790	10546	3624	4039	5832	2,6

22472	30836	34864	47560	10118	13792	23345	10,3
36983	33821	60594	91193	27286	46489	44592	19,7
15622	24988	11693	22015	38004	73470	25779	11,4
4367	9744	1890	4284	11519	15514	6597	2,9
1777	3521	1089	1971	4197	4820	2435	1,1
3450	6579	8289	9473	1626	1742	4635	2,0
3831	3466	7672	9446	2080	2287	4640	2,0
5826	8178	7534	9545	5578	7273	6483	2,9
7056	16215	7333	11606	13614	25935	11149	4,9
198373	326011	206315	308160	191882	305728	226808	100,0
632	462	534	558	353	830	574	7,8
1268	545	1003	608	534	1314	917	12,5
2831	1617	2140	2978	1612	2935	2519	34,3
2704	546	14945	4060	514	1510	3336	45,4
7436	3170	18621	8203	3012	6589	7346	100,0
1951	1232	1218	2067	2827	2087	1864	1,2
5468	8513	4539	7570	13086	10583	7561	5,1
9933	6768	7538	12011	11231	7748	9496	6,3
2276	959	2617	4141	2228	2743	2371	1,6
3771	4749	2301	5120	7803	9249	4825	3,2
14006	24798	9770	16348	15607	19267	14978	10,0
12401	13267	15519	24580	11587	15209	14157	9,5
10656	6099	13800	14542	5741	6679	9005	6,0
41149	24819	66049	74414	22725	31929	39343	26,3
19467	40979	19928	29162	30717	56390	27987	18,7
5126	18290	3408	6515	8248	13228	7822	5,2
1232	737	2394	2804	1661	1983	1713	1,1
1688	1626	2773	2978	2255	2594	2140	1,4
1990	2056	1992	2439	2383	3512	2104	1,4
3434	4797	2681	5936	6131	6191	4336	2,9
134547	159688	156528	210628	144229	189393	149704	100,0
860	750	602	873	395	448	662	2,5
1734	1714	3290	3804	1322	1359	2076	8,0
10546	13831	15977	20453	4707	6784	10681	41,1
3196	4441	847	2175	1838	1936	2158	8,3
1821	3086	4142	4691	844	943	2312	8,9
1732	1672	3782	4620	1005	1055	2206	8,5
3878	5527	1166	2644	1556	2768	2578	9,9
3585	896	4649	8212	1071	1929	3309	12,7
27353	31917	34454	47473	12739	17222	25981	100,0
9988	3383	5041	9596	13369	17088	9246	6,8
8942	14184	5516	8497	8197	13371	8746	6,4
91371	51050	86576	128167	45416	81708	78426	57,3
3947	5817	2972	2826	5229	4003	3938	2,9
7289	6767	5067	7253	4391	9085	5979	4,4
13121	10036	8952	14805	13985	18182	12226	8,9
2996	2850	1917	2557	5272	7862	3510	2,6
15680	17185	12305	17425	12657	18915	14783	10,8
153334	111271	128345	191128	108517	170212	136855	100,0
58782	43777	33242	42155	24099	63797	39671	56,2
2902	3878	2309	1769	938	1511	2126	3,0

5676	9695	9756	6658	2358	3349	5571	7,9
3340	3107	1621	2201	1467	3506	2434	3,5
7001	3634	2954	4086	3129	5820	4518	6,4
24173	9316	8462	13530	16498	24753	16222	23,0
101873	73405	58346	70400	48487	102738	70543	100,0
72485	130599	35999	57094	36920	38895	64706	1,2
58864	248937	23970	41460	24656	19282	68514	1,3
60692	104956	75552	99259	46635	85034	72627	1,4
2641335	2984902	1015039	1515170	976730	652752	1639038	30,6
19102	19673	16233	29409	5984	8205	16377	0,3
115694	317326	38272	62019	46634	37125	97570	1,8
85899	62847	43182	56944	43871	73801	56063	1,0
516842	1120761	872450	946380	283661	689612	655333	12,2
4150710	2549546	1693261	2297059	1805812	1149583	2297693	42,9
5570	7594	5792	7837	1696	4364	5314	0,1
34824	77698	14227	18791	15185	8452	26934	0,5
58954	10732	152429	179948	3566	13150	67426	1,3
35392	53683	48721	70090	34588	190898	59592	1,1
14043	45721	20477	28649	14474	50331	23935	0,4
15741	50689	24506	30701	10846	29124	22832	0,4
95230	107677	38833	55197	44555	21148	61091	1,1
12599	38897	6217	8808	7196	2994	13470	0,3
3200	1648	10945	10979	1159	1923	4711	0,1
6459	2968	11937	9625	1838	4014	5806	0,1
7043	5273	19844	17955	5862	20507	11009	0,2
9347	11488	10641	17759	14393	72861	18221	0,3
2827	6901	3690	5009	3600	9373	4410	0,1
3761	11000	5928	6672	2978	6765	5404	0,1
52514	121030	24641	45402	26595	19100	51642	1,0
8079129	8092548	4212784	5618217	3459435	3209292	5349715	100,0

PM	PM	PM	PM	PM	PM	PM	PM
SK-MEL-28 (I	Mel-HO (P9)	SK-MEL-28 (I	Mel-HO (P10)	SK-MEL-31 (I	A375 (P9)	A375 (P10)	G-361 (P5)
12908	64508	16025	97795	6035	20798	22803	58325
76811	632591	254337	1054541	132209	90600	112023	529125
30393	31863	33851	44097	41709	19471	31406	52323
17272	70735	18997	84048	10925	10993	9017	73552
177561	1266019	621863	2284275	207779	280066	300199	1949467
130230	552344	365805	780629	431473	96815	139333	547622
67471	711709	310965	1039825	140478	160801	208724	1025642
901998	2716154	2348037	3857984	1404416	1716740	1685139	3365960
9259	24096	24591	41591	52912	33519	46769	33101
16335	34228	52856	57317	34405	53241	38493	111615
13282	25630	33963	21233	94225	16819	12779	17692
18828	76059	78107	151500	110914	59915	62475	100462
187121	1313753	682407	1492166	314077	851374	834422	1251844
82644	200121	307624	474338	174104	781161	534210	334436
80278	24990	30449	41150	112819	65259	32775	25005
5811	8272	13947	13474	34608	12971	9563	10699
10385	52191	31719	79823	10848	23501	15167	50639
2130	3547	3360	5611	3262	5992	4642	4695
32078	3889	11145	15082	19575	19683	7026	6655
7352	6180	9710	8061	23448	8560	5614	6801
3513	2468	3855	3888	9285	3811	2921	2915
2408	2478	1828	3651	1768	3286	2805	3577
1886066	7823825	5255439	11652078	3371273	4335377	4118303	9562154
4424	56664	19832	103652	6609	3954	9959	32292
18737	135854	46680	202770	17732	19534	27571	127789
35399	286428	156930	404642	37063	12005	45456	131268
68167	837574	341610	1429569	100673	52185	113683	498929
2059	71578	29795	125001	9895	2985	14121	40161
128785	1388097	594847	2265634	171973	90663	210790	830437
1013	1561	1406	1628	541	891	861	2371
37149	9855	3221	1618	2842	41536	21576	1849
8250	2126	560	427	635	8259	4096	801
2498	819	3302	1128	1770	2819	3068	2220
2620	2192	3964	3071	4506	2655	3281	3176
42310	12758	80230	21860	64881	33833	26462	67339
4964	2427	8934	3991	8716	4199	3116	16414
3546	5747	6439	5964	6758	3098	4674	6370
5563	2216	9978	2045	4764	9459	7452	5842
415	901	929	1276	759	731	717	1229
1386	1666	2619	2889	3845	2675	2081	3267
3245	2291	4431	2827	6696	5997	4048	3674

2898	2034	5285	3093	6278	2981	2941	4048
1160	780	1667	834	2407	2014	1559	864
4230	7109	11007	8773	23001	20553	16196	8867
20519	22980	40357	32052	92581	15637	15216	37512
4930	3068	10177	3219	16288	10651	10013	7838
512	476	690	497	1494	435	537	892
146195	79447	193790	95564	248222	167533	127033	172203
1842	8289	3732	9436	6167	3777	4001	6231
1499	2792	2782	3672	2863	4738	5305	4410
3341	11081	6514	13107	9030	8514	9307	10642
2285	8859	3037	10315	1599	3503	3001	9437
7560	57427	14752	55560	5666	7911	6811	31323
5743	27589	11589	31141	3182	12191	11960	26509
15587	93874	29379	97015	10446	23605	21771	67269
379	2401	684	3843	262	557	861	2200
1309	2778	1514	3613	4347	1524	1715	2399
398	1330	435	1232	287	529	687	1115
420	1500	521	2061	563	461	493	1067
738	188	2141	109	406	7893	7481	288
3698	2694	932	2318	1429	1170	1392	4565
6942	10890	6227	13176	7294	12134	12629	11633
3179	1094	722	2012	982	1678	1197	3951
2493	1370	1050	2885	1383	1990	1551	10995
4031	3019	1997	2348	2105	3621	4236	21115
2281	2674	1795	3706	2132	1728	1544	14050
6720	6815	5548	7491	5592	7171	8646	125233
8797	7321	7448	7386	5396	14584	15353	111107
2346	2490	2463	3155	3411	1420	2345	19136
6043	3936	3460	2692	3639	3248	4258	24551
1419	1994	1114	2356	1639	1149	1206	7113
6865	11338	7040	10614	6489	5935	6445	137525
16663	20294	27185	23822	12214	29080	39696	553040
13013	14468	26628	11959	14327	28222	52128	191421
2905	2398	2581	2385	2640	2027	1952	17963
5895	3613	6918	3568	4654	5939	7089	86032
4121	2752	4434	1874	2924	6497	6123	33867
1191	1086	955	1002	1644	1066	1251	2849
5922	9776	8088	7398	8367	5000	6933	62094
24422	45487	53385	33232	30899	26614	51519	780893
22896	38648	84792	23390	33073	62752	177372	847890
9519	12497	25624	8464	14678	46353	79798	151865
1406	1093	1215	1279	1537	894	1394	6560
4042	3224	6303	3054	3368	4555	7055	68080
3982	3665	6998	2947	3745	11383	12050	72510
2131	2305	2475	2781	2888	1950	1350	2822
5428	7898	7992	6923	9022	4781	7363	9790
10004	15841	16602	11357	20615	8180	15086	26064
20106	41501	43063	22552	41086	18497	40032	279083
30903	71847	106161	35667	67343	58813	155413	739884
13297	17946	41380	13129	23328	87528	135007	336170
4146	4354	9902	4228	6947	43573	39476	35636
1654	2021	3558	1355	3116	2200	3008	18917

2450	4519	7919	3215	5151	5776	7951	49250
4621	6222	6607	5183	9349	4231	6633	10651
5775	10459	10068	7168	14309	6031	9719	16902
9010	16737	21115	12174	25258	9068	20839	44358
18563	45904	60263	27847	58163	23168	65342	221941
9746	17812	27262	12961	20627	27186	53079	185340
3436	3834	8012	3465	4794	25207	26723	40787
1111	1119	3319	917	8453	2669	2419	7510
1133	1247	2155	1069	6409	2205	2375	8812
1206	1428	2306	1099	4705	2040	2530	7486
1516	3112	3752	2358	3899	3370	4523	26795
1522	2429	3591	2144	2624	6563	6670	20767
1010	1132	1752	1131	1178	6647	7017	7369
689	431	997	496	2956	931	1156	6226
610	497	960	478	2180	933	1181	4053
498	378	689	395	1121	739	982	2333
719	1034	1269	805	1027	1428	1847	6196
1037	1228	1592	1048	1281	3868	4892	7889
1011	871	1582	855	969	8881	10034	5447
601	563	1064	650	784	3619	4814	3798
314084	485723	685150	352469	516420	642989	1128602	5482115
3927	11596	4199	17438	2679	6048	5265	11569
4605	7468	7498	10930	4825	5823	7506	9771
6136	21253	14033	29683	6130	13723	19279	22633
8407	12239	17198	16025	11510	11534	15452	11487
3294	12568	6051	12241	3390	6816	9045	13656
1608	4442	3403	5052	1944	5605	6143	3292
43688	43328	31410	48832	50617	20290	44232	68884
71665	112894	83791	140201	81095	69837	106922	141292
3588	15373	7237	11740	1918	2928	3518	26624
6235	89187	20783	54574	3344	8082	11457	81783
15982	55095	34167	49910	9797	17816	20963	76829
1864	4414	5245	4203	3246	2290	2546	5368
3202	12396	7617	11228	2978	5737	5829	11403
25635	126382	59560	123819	40418	52207	54748	74045
10503	24274	20050	24064	19337	11383	14838	21875
5874	8538	9518	9778	26561	15923	17071	8162
5262	6180	6801	6343	5751	5779	4756	7458
2321	11949	5413	11380	2638	4067	2746	10112
10337	23541	16194	22079	18005	12816	12814	18242
1797	1648	2553	2413	3125	1872	1611	2484
92600	378977	195137	331532	137117	140901	152896	344385
3489	10135	4704	14431	1032	2625	2224	36357
1212	2613	2034	4461	1188	1002	920	3803
29807	74175	56832	122731	12980	29249	20764	168283
878	850	1126	1212	512	831	534	1896
5005	2444	7345	4309	5243	4048	2927	4651
9685	7744	14439	15618	19023	12239	8863	12636
8486	26807	14998	39113	4488	7104	4338	31835
7481	17118	13954	30585	4378	17856	10799	29158
8632	7974	14364	15482	6843	8096	5972	15043

8116	8874	13245	20268	17448	11453	7928	15120
4962	3613	7523	7930	29816	14824	9146	6577
2076	1917	3000	3025	6978	5616	3658	2879
828	5039	1575	4494	1310	2045	1154	6282
806	2416	1390	3751	1055	1924	1529	4315
1898	5871	3700	7615	2048	1547	1343	5589
2796	2331	3881	5878	4526	6960	4153	4462
3679	3749	5562	6968	9165	7633	5126	7111
1324	1662	2040	2105	5761	1478	1594	2514
101159	185333	171710	309976	133796	136529	92971	358510
367	2824	1156	1507	384	336	582	2204
680	6939	5341	4796	530	521	955	11122
1029	6386	3974	4808	1408	1804	2386	6864
607	3560	3755	899	962	504	1623	2876
2683	19709	14225	12009	3285	3166	5546	23066
878	3442	2432	6536	779	909	1303	3629
3335	21814	9718	30216	2139	2460	3783	21280
4606	19240	16487	32256	4972	6836	9596	15880
1649	2224	4305	3816	3160	2069	2203	6267
1745	3925	5473	6755	2756	2199	2962	7077
7516	41831	26644	64264	8141	14326	21337	47926
5513	37366	20149	62611	6644	21484	28289	19029
6763	11691	20536	21348	13538	9043	8497	25968
12676	11743	37566	25125	42826	29719	27298	53967
7886	8309	20117	17844	17655	15392	15372	25652
2948	10191	8266	14574	4471	6254	6905	10520
2225	2784	5746	3795	3886	2658	2777	7494
2507	2022	6089	3637	7630	4343	4413	7938
1653	1114	3213	1910	4772	2321	2420	4141
1239	3726	3133	4591	3235	1632	2228	3903
63140	181421	189874	299276	126606	121645	139384	260669
2387	1203	3099	2238	2767	2016	1310	2231
3320	2673	5957	5806	2371	3608	2680	5932
3603	4197	6359	8963	7901	5396	3757	6713
366	1955	1123	1561	309	584	629	2144
1113	2115	2057	3710	1067	824	850	2346
1394	1086	1941	2005	2064	3447	2581	1685
293	916	615	917	518	424	390	1055
1871	2467	4962	3149	1212	1884	1873	7378
14348	16611	26114	28351	18209	18182	14069	29484
3147	13699	12667	10899	4070	2573	4816	20510
3229	20683	9188	14264	3826	2906	3621	12788
17985	79599	62024	68617	51276	18288	28379	53572
6264	4650	4111	5097	2701	3648	3813	4553
1422	4579	3261	4107	2101	1107	1146	3699
6088	15956	12178	14550	7046	9260	10660	18225
1328	2292	2050	2299	3470	2234	2382	1945
7084	19829	12999	17625	9490	15186	16858	17223
46547	161288	118479	137459	83980	55202	71674	132517
4676	29973	12023	31945	9085	7209	8096	52009
1000	1625	924	1097	713	809	1157	2057

1858	6857	3698	5951	1369	2860	3193	8372
650	3452	969	2641	1039	1216	1445	2469
1452	3383	2090	3386	1762	3098	3338	3996
5899	8937	8818	12373	7656	16250	17847	13531
15535	54227	28522	57393	21622	31442	35075	82434
368052	58403	27412	24709	50257	137906	132418	65095
139939	25231	20669	16695	35367	20052	28965	38475
69466	33377	30455	24161	39354	28274	32280	44105
1933056	1236349	895512	1031290	908689	1032320	1050652	1202322
25604	7917	8519	7258	11878	9215	9419	9487
166957	51027	31137	27691	46778	37974	43837	54250
44861	21171	29939	18473	30505	25793	32165	18135
559381	209636	338146	120785	259918	456272	507376	277996
2127921	2277084	1384491	1858182	1207909	1619151	1724554	1986703
7217	5555	3419	1673	4275	5205	4823	9906
50095	13578	7624	6944	9676	10859	9155	13510
7706	2423	15674	1019	65571	4840	3679	2289
3160	1803	7025	1153	11859	3346	2695	2017
4176	2286	3827	1842	5232	3808	3549	2761
12738	11857	10102	7254	8708	19321	18536	15898
119130	58125	27873	31676	26468	47841	45375	60422
43541	9689	4739	4191	4751	7293	4923	15333
3436	1695	3291	828	9932	2793	3016	2090
2408	1676	5128	590	23210	4297	3582	1935
1701	1169	3279	1191	10342	1850	1743	1637
971	558	813	490	2311	712	866	564
1426	728	553	597	1138	2039	1690	984
5391	3804	2314	2640	2579	8120	6623	3632
103363	38192	25576	20264	18957	27356	22384	62593
5801698	4073335	2887518	3211597	2795663	3516635	3694304	3892141

PM	PM	Primary Melanoma			MM	MM	MM	MM
G-361 (P6)	SK-MEL-31(P	Mean	% Class		Hs294T (P7)	A2058 (P5)	SK-MEL-3 (P5	RPMI 7951 (P
66022	3794	36901	0,6		37500	41212	77057	22359
337330	122460	334203	5,8		259193	356093	749498	138604
51613	40331	37706	0,7		58371	52285	47366	50527
83679	5877	38509	0,7		39634	48820	39865	17568
1259032	231295	857756	14,8		1369854	1311320	1396343	685090
371346	372649	378825	6,5		295138	389814	430856	186060
575246	146616	438748	7,6		527820	799565	610906	386652
2554550	1687940	2223892	38,4		2986075	3340270	3500350	2478494
22796	44673	33331	0,6		48617	47197	42865	41769
63682	24666	48684	0,8		75012	60922	43416	54494
12467	45704	29379	0,5		57294	43954	31994	40138
48904	72382	77955	1,3		161728	147379	73924	117342
860445	224887	801249	13,8		1112394	1851582	1468718	1016063
179487	148490	321661	5,6		1098779	517726	809056	536551
69606	72959	55529	1,0		63888	50356	37579	47487
7693	21689	13873	0,2		38977	16279	11253	21524
25715	13112	31310	0,5		61842	66586	72998	41621
3332	2175	3875	0,1		8596	7152	4478	7768
13686	19075	14789	0,3		31175	16639	13725	14012
5670	10849	9224	0,2		15647	13359	8883	10502
2991	4144	3979	0,1		7620	5620	3393	5377
2834	916	2555	0,0		3944	5040	3927	4553
6618126	3316682	5793932	100,0		8359098	9189171	9478451	5924555
17869	6187	26144	4,2		22687	22611	30123	29314
82107	14834	69361	11,0		76975	65473	78860	110075
77381	37102	122367	19,4		95445	134296	100792	173431
271563	75638	378959	60,2		313098	722411	470759	633167
23495	8433	32752	5,2		42281	93242	51616	106642
472415	142194	629584	100,0		550486	1038033	732149	1052629
1516	353	1214			691	663	869	926
		1214	100,0					
34849	2104	15660	9,6		3961	4214	3277	3517
6249	563	3197	2,0		669	985	721	704
2945	1587	2216	1,4		3679	2466	1064	2998
3851	2761	3208	2,0		5920	4358	3881	4906
69399	54052	47312	29,0		120516	71998	75210	105775
14399	5565	7272	4,5		11147	7057	8284	12306
5027	4500	5212	3,2		6506	6983	6731	7333
6056	5479	5885	3,6		7195	5097	9357	5095
1137	473	857	0,5		1262	1430	575	2616
2548	2493	2547	1,6		4689	2825	1621	4018
3579	3956	4075	2,5		16481	6083	2405	11408

3824	5125	3851	2,4	7148	4180	1727	6686
809	1828	1392	0,9	4592	2001	2000	4751
4353	15178	11927	7,3	17373	13439	12193	13201
34556	80437	39185	24,0	68761	61487	22697	48644
8103	12910	8720	5,3	37007	14742	3161	20062
676	991	720	0,4	1603	1110	400	947
202361	200003	163235	100,0	318508	210454	155304	254965
5185	3099	5176	60,2	7378	8863	6414	10561
4246	1851	3416	39,8	8055	4418	2661	9146
9431	4951	8592	100,0	15434	13281	9076	19707
7074	633	4974	11,8	3497	5084	10520	6335
26010	2746	21577	51,1	13756	29166	40717	14505
25402	1693	15700	37,2	18251	19473	25798	19970
58487	5072	42251	100,0	35504	53723	77035	40810
1323	183	1269	13,6	665	2253	1308	1333
1723	1317	2224	23,8	2780	3514	1636	3741
1049	235	730	7,8	503	1135	618	601
812	363	826	8,8	579	1012	888	424
259	137	1964	21,0	1623	761	294	2511
4057	1179	2343	25,0	1531	2336	1011	2187
9222	3414	9356	100,0	7681	11011	5754	10797
5080	481	2038	0,1	1349	1529	1098	810
12251	1272	3724	0,3	1025	2961	2303	1083
22409	1858	6674	0,5	3453	5193	5125	2088
14491	1468	4587	0,3	1465	4565	2673	1781
133611	3969	31080	2,2	6121	16501	14145	4522
93753	4022	27517	1,9	12915	13810	17893	6032
18133	2986	5789	0,4	1894	5236	3984	2337
21993	3089	7691	0,5	3670	5718	4577	3328
6895	983	2587	0,2	1240	3595	2066	1224
127541	4556	32435	2,3	7293	26336	14840	4614
425430	9769	115719	8,1	34579	69980	69365	10879
168669	10319	53115	3,7	53818	31931	41510	16105
15706	1919	5248	0,4	2010	4974	3025	1955
67669	3087	19446	1,4	5959	9293	6242	3661
26831	1894	9132	0,6	4422	4823	4167	2546
2601	980	1462	0,1	1114	2201	1834	1023
54551	4948	17308	1,2	8148	18420	14332	7425
545032	22267	161375	11,3	52779	147689	103945	28103
714274	29626	203471	14,3	132601	156843	161315	31385
114305	12154	47526	3,3	53277	31180	37458	16229
5605	862	2184	0,2	1362	2864	1502	1041
50003	2120	15180	1,1	5348	10668	5337	2508
51466	2588	17134	1,2	8215	8892	7723	3174
2897	1959	2356	0,2	3250	3442	5753	3047
9275	6047	7452	0,5	9789	11011	18382	10315
23557	12899	16020	1,1	20075	23443	37743	20910
199566	23057	72854	5,1	43851	102954	78952	35053
660552	39804	196639	13,8	116763	308464	221267	48461
216284	13523	89759	6,3	69616	77766	74171	18685
25964	4449	17867	1,3	24221	10375	12798	8052
13069	1412	5031	0,4	2494	5265	3657	2363

30711	2710	11965	0,8		6036	12163	8662	3983
9503	5894	6889	0,5		8050	9460	12770	8250
15332	9021	10478	0,7		11993	14535	18480	12049
36648	14767	20997	1,5		23179	30087	29695	20816
158917	36561	71667	5,0		59778	151489	82196	52131
115918	11611	48154	3,4		33804	67815	41353	16100
27815	2834	14691	1,0		16240	13744	10779	4307
5782	2892	3619	0,3		2727	4342	1772	1315
6700	2047	3415	0,2		2886	3749	2218	1402
5482	1408	2969	0,2		3239	3788	2433	1874
15690	1422	6644	0,5		4375	9482	6032	2289
16138	1295	6374	0,4		5383	8216	4848	2347
6684	679	3460	0,2		3520	3357	2220	1093
4549	1117	1955	0,1		1171	3325	787	453
2316	771	1398	0,1		1054	2168	747	524
1810	403	935	0,1		796	1418	618	472
4938	506	1977	0,1		1679	3601	1535	726
6629	658	3012	0,2		2710	4158	1800	896
4934	554	3514	0,2		3998	2813	1516	811
3589	445	1993	0,1		2298	2955	902	642
4329549	327963	1426506	100,0		889031	1480587	1210547	433218
11330	1753	7580	7,8		6372	12174	14044	5608
9483	2875	7078	7,3		10056	9257	7362	12018
19401	3121	15539	16,1		15408	33859	19391	19653
11299	6328	12148	12,5		18463	14821	13933	18584
11082	1956	8010	8,3		6096	13339	9538	7808
3789	1134	3641	3,8		5139	6990	4209	7675
25331	51530	42814	44,2		44875	54262	50021	65896
91716	68698	96811	100,0		106409	144701	118498	137241
21433	2386	9674	4,4		9087	7609	15154	3264
69926	3601	34897	15,9		15531	28220	58461	4877
64800	10006	35537	16,1		39046	42599	65826	14871
3840	4183	3720	1,7		8876	3776	4183	4285
9132	3249	7277	3,3		7238	6135	9037	2780
66344	40262	66342	30,1		85690	92220	109466	49026
16603	20972	18390	8,4		25327	23557	25527	16977
6067	22746	13024	5,9		30708	16797	8701	21819
5824	6123	6028	2,7		10648	5271	6242	4876
8406	2982	6201	2,8		6132	7362	12782	2903
15308	18570	16791	7,6		19293	18919	18121	16431
2280	2230	2201	1,0		3704	2957	2869	2114
289963	137307	220082	100,0		261281	255421	336370	144224
31554	1176	10773	5,5		5676	4877	21724	1555
4139	1107	2248	1,1		1656	1864	5495	534
146008	14811	67564	34,5		58136	47529	212331	13416
1505	507	985	0,5		1152	1065	1727	482
2679	9259	4791	2,4		10781	4890	8180	4887
9364	22462	13207	6,7		36551	17100	26718	19420
25028	5260	16746	8,6		13574	17196	58865	4998
26946	5291	16357	8,4		20083	27509	72023	6482
13218	13577	10920	5,6		34530	13394	34341	18824

11452	20617	13452	6,9	29617	15911	36090	20943
5703	30419	12051	6,2	24521	15211	14379	17549
2824	7585	3956	2,0	9538	5330	4774	5402
5524	1613	2986	1,5	2689	3469	6377	2075
4798	1454	2344	1,2	2516	4969	7548	1492
4516	3484	3761	1,9	7305	5556	12627	3986
3602	7168	4576	2,3	15241	7653	16605	9603
6132	10639	6576	3,4	12391	10979	13375	11252
2309	4785	2557	1,3	4136	4757	3744	3222
307302	161216	195850	100,0	290093	209260	556922	146125
1758	535	1165	11,5	526	623	735	664
3794	390	3507	34,7	1228	1149	2220	696
5040	2357	3606	35,7	3508	2037	4010	4410
1735	1639	1816	18,0	2104	1166	745	2697
12327	4921	10094	100,0	7366	4975	7710	8467
2616	760	2328	1,4	2970	5271	3948	3831
13429	1996	11017	6,6	7746	22566	18947	8027
11865	5930	12767	7,7	16623	38332	20423	21795
3072	2958	3172	1,9	14348	2650	6113	4570
4298	2640	3983	2,4	11413	6159	5805	5533
33242	6746	27197	16,3	26433	68741	63639	30568
17367	7209	22566	13,5	18652	56342	50723	66018
11800	13203	14239	8,5	32546	20604	29020	17406
26937	34792	30265	18,2	72758	19359	61593	40845
15361	18285	16187	9,7	35929	13170	26252	26859
8135	5797	7806	4,7	11342	14656	19015	15128
3528	6269	4116	2,5	8993	5296	9107	5775
4523	9917	5302	3,2	9141	4083	9936	7530
2068	4238	2785	1,7	4253	2555	5163	3462
2643	3555	2989	1,8	4604	3850	4309	4871
160882	124295	166719	100,0	277752	283635	333992	262216
1201	4411	2286	10,6	4969	2415	4074	2564
5100	4899	4235	19,7	15929	5531	14984	8150
4678	9755	6132	28,5	12657	7479	15134	8927
1716	358	1074	5,0	1252	2929	2004	1191
1621	1654	1736	8,1	4070	3478	5896	2078
1662	3880	2174	10,1	8554	4668	4728	4833
869	482	648	3,0	954	1628	1587	877
5579	2049	3242	15,1	6329	6442	7182	3485
22425	27488	21528	100,0	54714	34570	55589	32106
12075	4824	8928	9,0	12073	10256	10521	8668
6986	4127	8162	8,2	6984	14853	8649	7051
33952	57748	47144	47,6	64031	69862	48885	45415
4424	2484	4174	4,2	2551	3543	4953	2048
2667	2688	2678	2,7	3246	3334	3929	4038
14949	7204	11612	11,7	14559	19259	12998	12479
1497	2803	2230	2,3	3382	3513	1712	3054
15146	9421	14086	14,2	18533	27640	18318	18706
91696	91299	99014	100,0	125358	152259	109965	101458
39213	9985	20421	48,3	32818	28911	48540	66029
1782	915	1208	2,9	1328	1768	2340	1749

6977	1880	4301	10,2		5148	6256	14487	10338
2133	738	1675	4,0		2378	2471	1922	5178
3925	1663	2809	6,6		6126	3989	3013	16245
17295	10162	11877	28,1		33958	19665	8996	40752
71325	25344	42292	100,0		81756	63061	79299	140291
312735	88790	126578	3,2		54267	73047	39674	51459
29876	63269	41854	1,0		26861	44335	23325	32193
59611	38274	39936	1,0		30816	48947	43437	25827
1545828	1620903	1245692	31,0		816128	1347666	1084434	1108620
13047	12857	11520	0,3		7947	10508	7858	6678
63903	118656	64221	1,6		31929	57376	56138	43172
39326	33475	29384	0,7		21481	30701	36693	19343
668634	286374	368452	9,2		312009	305611	460166	164496
2602617	2216888	1900550	47,3		1039029	1474105	1631750	1451934
6391	11421	5989	0,1		8002	7243	3829	4020
16010	21694	15915	0,4		9417	14802	10216	13200
6579	54631	16441	0,4		4358	2098	15977	1357
3299	11084	4744	0,1		5513	1924	5484	1744
3927	4690	3610	0,1		7119	3332	6039	2453
19818	9619	13385	0,3		15607	14072	20450	11185
81900	53056	55187	1,4		23400	41290	33934	31178
12138	11196	11779	0,3		4538	10012	5602	5811
2657	12959	4270	0,1		5417	2171	4506	1483
3596	16888	6331	0,2		5397	1229	7805	1153
1865	5980	3076	0,1		2779	1613	3553	1250
841	1481	961	0,0		1408	735	1027	880
1195	1098	1145	0,0		1678	1728	2061	1187
5187	2593	4288	0,1		5013	5125	4847	3978
41206	37917	39781	1,0		17445	32628	31266	24435
5542185	4735793	4015087	100,0		2457560	3532298	3540070	3009036

MM	MM	MM	MM	MM	MM	Metastatic Melanoma	
HS294T (P9)	SK-MEL-3 (P6	A2058 (P6)	RPMI 7951 (P	Colo-800 (P5)_	Colo-800 (P6)_	Mean	% Class
29000	60132	48635	26927	58945	61671	42899	0,5
248314	825282	387028	159166	572890	499937	383331	4,9
47992	45718	63326	54720	50279	46457	49845	0,6
30960	32621	44024	21057	67490	72423	37987	0,5
841412	1370150	1190260	811563	1427266	1329769	1094195	13,9
233427	508519	294053	189987	360286	348777	313143	4,0
545339	678518	761558	479912	685681	672792	588142	7,5
2712947	3733089	3360352	2485634	3063273	2495320	2904301	37,0
35126	53089	67628	42001	19828	14285	41761	0,5
54542	48839	81891	53199	46915	33487	53318	0,7
30702	23427	48147	30992	25683	25228	36119	0,5
141420	76823	171240	121582	128612	147945	124264	1,6
1318803	1518074	1661370	1276795	1307503	1313425	1335677	17,0
1094535	905958	699731	540998	332283	255000	676995	8,6
36166	36677	73891	36582	21213	18805	43521	0,6
36419	12265	19072	21506	34564	34576	23544	0,3
58732	69549	41157	56325	49966	36720	54309	0,7
6392	5618	7779	9591	3538	2540	6306	0,1
14358	12794	20184	8614	7685	5962	14097	0,2
8705	7745	23711	7858	5251	5270	10900	0,1
5898	2911	8652	4070	6554	5832	5450	0,1
5027	4459	5112	7034	3201	2627	4407	0,1
7536220	10032257	9078800	6446114	8278906	7428847	8175242	100,0
16467	39856	19455	29989	39032	41304	26487	3,2
57496	62385	52173	147365	96156	100006	78761	9,4
64216	144655	80623	179431	126339	128857	115834	13,8
331833	598140	480911	842687	361059	359481	492881	58,7
52452	64478	71894	158530	65504	58227	72375	8,6
522464	909513	705057	1358001	688088	687876	824430	100,0
2339	2309	710	929	789	1549	1178	
						1178	100,0
2967	3002	3863	3428	4589	4146	3696	1,5
584	634	961	631	860	813	756	0,3
2753	1306	3442	2718	2004	1953	2438	1,0
4090	3677	5141	4436	3056	3022	4249	1,8
102565	75942	86364	96674	108767	85397	92921	38,6
9881	8866	9610	11098	13315	10630	10219	4,2
6584	5530	7951	6850	6801	5710	6698	2,8
5971	9239	5805	4870	14353	11182	7816	3,2
883	934	1679	2430	1196	873	1388	0,6
3829	1548	3512	3601	9860	7099	4260	1,8
10444	2071	9744	9091	9915	6424	8407	3,5

6184	1897	5711	5689	8639	6463	5432	2,3
2678	561	3808	1859	1296	1064	2461	1,0
17466	13407	13730	13737	12438	10499	13748	5,7
54903	19868	74694	43169	124013	81823	60006	24,9
23297	2647	20315	16040	9374	6402	15305	6,4
1219	426	1302	680	2006	1518	1121	0,5
256297	151556	257633	227000	332483	245018	240922	100,0
7228	4257	8107	6360	5722	8716	7780	60,9
5584	3175	6252	5412	3114	2397	4993	39,1
12812	7432	14359	11772	8836	11113	12773	100,0
3965	8166	4788	7613	5635	5587	6119	13,0
17765	31973	21824	15139	24243	25101	23419	49,6
15411	20507	19526	18463	9934	9219	17655	37,4
37141	60646	46138	41214	39812	39907	47193	100,0
739	1026	1433	1568	1058	1159	1254	16,2
2283	1222	2703	2316	2354	2467	2502	32,2
634	797	701	765	556	768	708	9,1
518	904	871	571	1003	806	757	9,8
1389	245	623	807	109	72	843	10,9
1287	859	3766	1879	1321	761	1694	21,8
6850	5053	10097	7906	6400	6033	7758	100,0
966	1052	1412	822	885	1433	1136	0,1
2111	1836	3425	1489	1676	1500	1941	0,2
3628	4735	5008	2635	2512	2069	3645	0,4
3487	2550	4277	2120	2879	2511	2831	0,3
14683	15413	13978	6525	8857	7115	10786	1,1
19969	19040	13266	7341	9658	7825	12775	1,3
4005	2546	6939	3776	2517	2234	3547	0,4
3922	2828	7514	4295	2577	2054	4048	0,4
2138	1693	2908	1706	1909	1824	2030	0,2
17135	17425	14058	6786	11801	10055	13034	1,3
89850	86268	42119	17420	37648	25633	48374	4,9
50061	45328	30081	18803	20500	12847	32098	3,2
4099	2241	5111	2401	2231	1789	2984	0,3
10273	5831	9760	4716	4026	3060	6282	0,6
5712	3506	5266	3302	2539	2034	3832	0,4
1381	1165	1543	1349	1268	1667	1455	0,1
15348	13702	11949	8950	10570	9431	11827	1,2
173813	129029	72225	38373	59751	38882	84459	8,5
257613	180370	101471	46791	57312	29627	115533	11,6
65571	42748	29188	20401	14766	10037	32086	3,2
2521	1351	2444	1230	1338	1014	1667	0,2
12204	5952	7176	3340	3812	2465	5881	0,6
14391	8733	7342	3770	5002	3315	7056	0,7
2932	4280	3153	2913	2329	2645	3374	0,3
9779	14184	10450	10086	7869	7512	10938	1,1
24337	30307	20934	20930	15506	13736	22792	2,3
114007	86652	52659	40754	42742	29644	62727	6,3
423687	283964	146406	58772	83321	43122	173423	17,4
195568	104385	51084	23452	20822	12991	64854	6,5
37060	16128	12983	8748	4995	4299	13966	1,4
7549	4338	3613	2144	3120	2066	3661	0,4

18942	10697	8142	4985	5671	3693	8298	0,8
8436	10129	8935	8271	5815	5596	8571	0,9
13274	15209	13456	13024	8843	7635	12850	1,3
34103	24988	26767	25385	16036	11512	24257	2,4
169032	105910	82547	56698	42784	27025	82959	8,3
145033	59656	38862	20010	17818	12269	45272	4,5
43770	14292	11414	5509	3580	2802	12644	1,3
4196	2552	2665	1763	1443	1089	2386	0,2
4691	2237	2653	2343	1799	1402	2538	0,3
9161	2649	2396	2694	2463	2072	3277	0,3
24483	9663	4336	3252	3605	2702	7022	0,7
24014	7327	5348	2945	2470	1938	6484	0,7
8846	2930	3344	1893	1101	914	2922	0,3
2006	1230	1831	1009	613	432	1286	0,1
1849	844	1477	1062	715	557	1100	0,1
2402	764	875	845	735	545	947	0,1
7022	2167	1644	817	1001	773	2096	0,2
8813	2548	2502	1193	1123	911	2666	0,3
8539	1845	2678	1080	586	589	2446	0,2
8335	1306	1897	687	654	497	2017	0,2
2136751	1418522	923511	531607	564710	379958	997076	100,0
5321	14309	9319	7145	6626	8723	8964	7,0
8146	7457	10103	11754	7927	5987	9006	7,0
14999	17847	22948	19632	18194	20731	20266	15,8
16167	11624	18013	16913	9582	7879	14598	11,4
9797	10157	9609	9095	7760	9820	9302	7,3
5279	4436	6790	7840	2957	2817	5413	4,2
106472	45883	74156	96658	35724	33246	60719	47,3
166180	111712	150937	169039	88769	89202	128269	100,0
8917	14041	7414	6577	12043	9908	9401	3,8
20942	62116	17827	6075	27708	27242	26900	10,8
39836	64159	36618	18485	52320	43259	41702	16,8
4249	4126	4868	3330	4521	3201	4542	1,8
7716	10116	6873	3460	11032	8942	7333	3,0
101238	114738	95866	48246	77798	63592	83788	33,8
19624	22318	24966	15494	20816	19533	21414	8,6
20457	7264	23780	15061	13046	11957	16959	6,8
10548	6113	6331	4286	11935	10786	7704	3,1
9543	13339	6116	3671	11345	9661	8286	3,3
13735	18328	16917	13015	17130	13390	16528	6,7
3921	2792	5031	1627	5912	5311	3624	1,5
260725	339450	252609	139326	265605	226782	248179	100,0
5319	21249	4320	2850	5814	5295	7868	2,8
847	4315	1800	571	2094	1472	2065	0,7
52511	213308	46758	17236	59655	49771	77065	27,9
895	1810	1256	514	813	692	1041	0,4
4914	6712	5128	5219	2381	2127	5522	2,0
16629	26311	17773	17882	21294	18324	21800	7,9
14031	58557	14642	7546	23872	20576	23386	8,5
17076	66867	25324	8883	14354	11330	26993	9,8
18657	28910	19733	14668	26321	23791	23317	8,4

14438	33202	19231	16951	22180	17895	22646	8,2
11952	14723	22716	15374	14316	13924	16467	6,0
6599	4745	6778	6360	8461	8521	6651	2,4
3133	7058	2895	2846	5597	5404	4154	1,5
2578	7928	4498	2411	3436	3118	4049	1,5
5709	12137	6205	3547	13303	13240	8362	3,0
7928	15272	12549	7641	9353	9028	11087	4,0
5405	12731	14516	8469	7596	6292	10301	3,7
2166	3495	6227	2577	4272	4087	3868	1,4
190787	539331	232349	141544	245111	214890	276641	100,0
1196	2387	526	467	820	1668	961	8,7
10043	5648	974	878	2324	3038	2820	25,7
5303	10211	3350	3241	3124	3533	4273	38,9
10540	4149	970	932	2520	3574	2940	26,7
27082	22395	5820	5519	8788	11812	10993	100,0
987	2920	7264	4076	3276	2801	3734	1,4
5256	16580	17463	8518	18370	18261	14173	5,2
7291	18583	48844	20129	15541	12069	21963	8,1
7585	5182	3582	2938	4801	4303	5607	2,1
10530	5820	5285	4799	11444	11846	7863	2,9
23569	62004	49247	33948	38838	33449	43043	15,8
8245	57338	51283	65081	13168	9161	39601	14,5
24483	26571	25964	15606	18091	17439	22773	8,4
63832	53413	25882	34035	41386	41274	45438	16,7
37781	24774	14807	26567	47717	49828	30368	11,1
13485	20793	10588	17617	23037	20033	16569	6,1
5697	9369	6078	4636	3817	3917	6268	2,3
5796	9415	5615	5068	4148	3856	6459	2,4
3253	4837	3588	3056	3275	3088	3653	1,3
5331	4152	3916	4503	7506	6616	4966	1,8
223121	321750	279404	250577	254414	237940	272480	100,0
2491	3131	2575	2562	1121	1149	2705	6,6
8006	11898	8941	6354	12100	10733	10263	25,1
6342	14828	8052	7824	9835	8283	9936	24,3
1328	1991	1935	1241	1935	1657	1746	4,3
3078	5635	3355	1863	6821	6951	4322	10,6
4278	4186	7393	3572	4958	5026	5220	12,8
1700	1684	1295	943	1989	1596	1425	3,5
5122	6606	9588	3227	2372	1655	5201	12,7
32345	49958	43135	27587	41132	37049	40818	100,0
9869	12525	10034	8313	15021	14439	11172	9,1
8289	11919	8751	6282	13819	13052	9965	8,1
63490	67657	57389	28740	56216	51526	55321	45,0
4048	4449	3886	2072	4448	4295	3629	3,0
3826	5336	2355	3177	5149	5452	3984	3,2
14324	13097	19508	12077	14543	13680	14652	11,9
3048	1661	3131	2794	2842	3124	2826	2,3
21278	21453	25466	22556	19886	19046	21288	17,3
128173	138097	130519	86012	131923	124615	122838	100,0
22281	42035	28622	33930	27278	23923	35437	49,1
1040	2372	1558	1500	1501	1239	1640	2,3

4045	12372	5234	4712	7985	6441	7702	10,7
1644	1226	1711	2139	3122	2680	2447	3,4
3221	2087	4650	6732	3414	2570	5205	7,2
20076	8073	30411	19355	8939	7271	19750	27,4
52307	68166	72184	68369	52239	44125	72180	100,0
60316	63473	71544	57954	30588	36757	53908	1,6
37510	31840	56140	41749	20357	24166	33848	1,0
75458	64706	54462	32893	23617	37039	43720	1,3
1406564	1076936	1600158	1418778	582888	900180	1134235	33,9
15116	10346	13684	9315	5663	7129	9425	0,3
45409	60695	62731	56673	24829	33487	47244	1,4
46432	30208	34441	23176	17046	23388	28291	0,8
521729	700666	264680	145604	152260	238108	326533	9,8
2106462	1353453	2213404	1954584	808468	1345892	1537908	46,0
6127	7051	3440	4952	2424	3190	5028	0,2
11184	10416	13884	10594	5070	6987	10577	0,3
14648	26878	1853	1002	2128	5106	7541	0,2
17476	8074	2033	1969	3290	8079	5559	0,2
15053	9577	3217	3290	5269	10543	6589	0,2
26215	32354	11736	11817	8979	12537	16495	0,5
41097	28173	46211	34737	16947	25807	32277	1,0
4538	5688	7027	5684	2827	5031	5676	0,2
7199	7634	1708	1076	2898	2754	3684	0,1
6414	10869	955	954	1193	1992	3796	0,1
3950	5037	1377	1211	1602	2031	2440	0,1
2944	1123	725	780	1548	2168	1334	0,0
3221	3159	1177	1250	1228	1207	1790	0,1
6327	7332	4155	3392	3681	3304	4715	0,1
21902	24434	26842	22606	11616	17899	23107	0,7
4503291	3580119	4497583	3846041	1736416	2754780	3345719	100,0

Table S2. Ultra-High Performance Liquid Chromatography (UHPLC) and Mass Spectrometry (MS) analysis settings.

**UHPLC**	**MS**
ACQUITY UPLC (Waters)	Quadrupole time of flight (Q-TOF) model SYNAPT G2 HDMS (Waters)
Pre-column: Acquity UHPLC HSS T3 1.8 µm VanGuardT (Waters)	Ionization mode: ESI positive and ESI negative
Column: Acquity UHPLC HSS T3 2.1x 100 mm, 1.8 µm (Waters)	Adquisition mode: continuun MS^E in resolution mode (FWHM≈20,000)
Column temperature: 65 ºC	Capilar voltage: 0.7 kV (ESI^+) and 0.5 kV (ESI^-)<br>Cone voltage: 35 V
Flux: 500 µl/min	Source temperature: 120 ºC<br>Desolvation temperature: 400 ºC
Mobile phase A: acetonitrile/H_2O (40:60) with 10 mM NH_4Ac	Desolvation gas: argon, 900 l/h<br>Cone gas : argon, 30 l/h
Mobile phase B: acetonitrile/isopropanol (10:90) with 10 mM NH_4Ac	Acquisition range: 50 to 1200 u.
Gradient:<br>0-10 min, from 40 to 100% B<br>10-11 min and final re-equilibration of the system, 100% B	Scan time: 0.5 s<br>Inter-scan delay: 0.024 s
Automatic injector temperature: 4 ºC<br>Injection volume: 7.5 µl	Colission energy:<br>Function 1: trap 6 eV and transfer 6 eV<br>Function 2: trap ramp from 15 to 40 eV and transfer 6 eV

Figure S1

	ESI +	ESI -
NM vs HeM	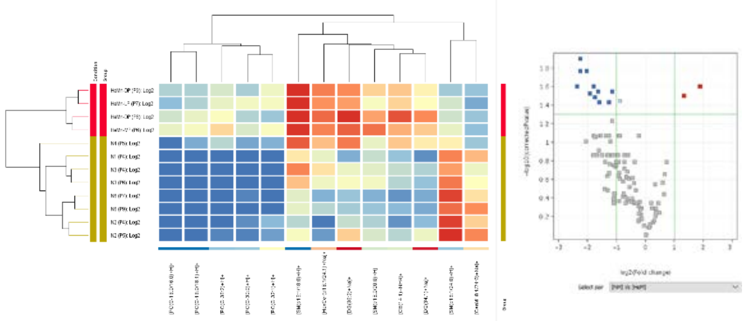	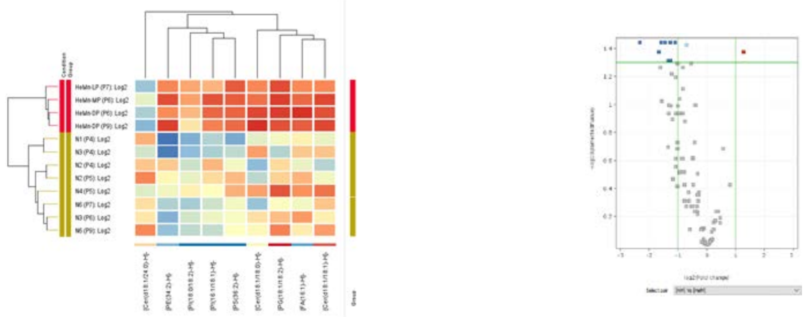
PM vs HeM	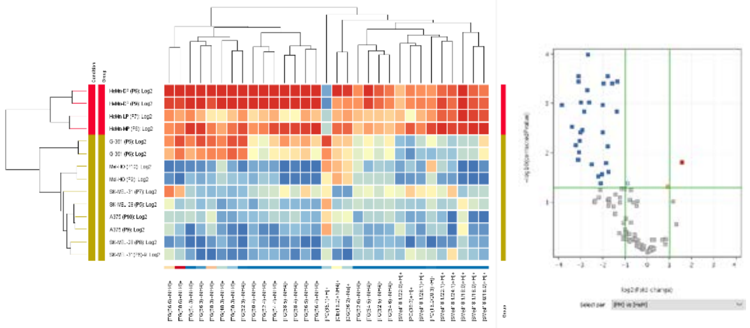	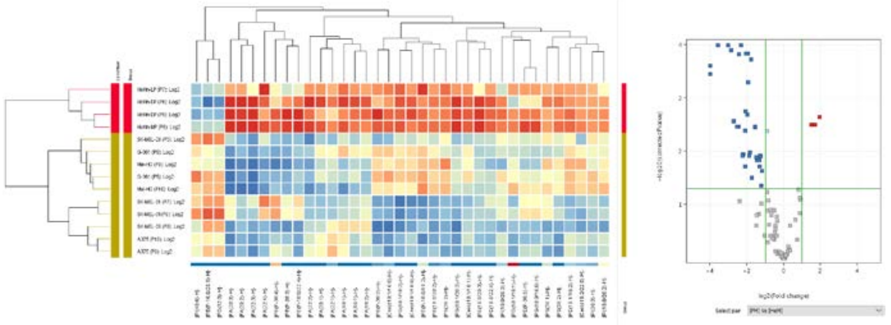
MM vs HeM	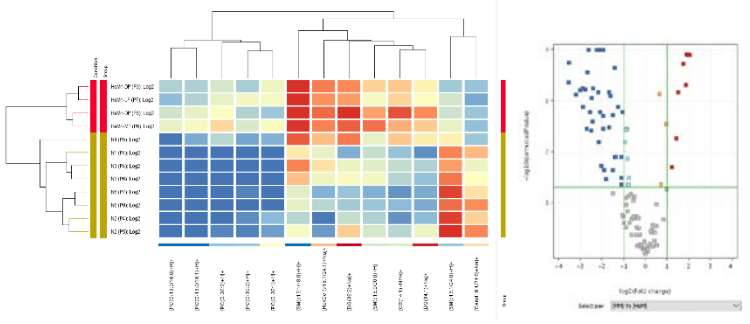	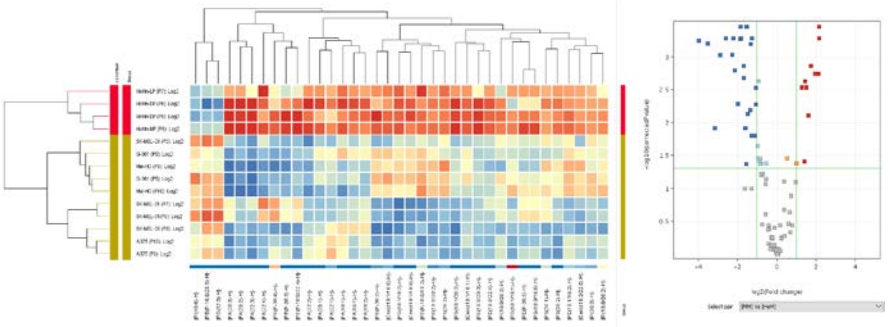
PM vs NM	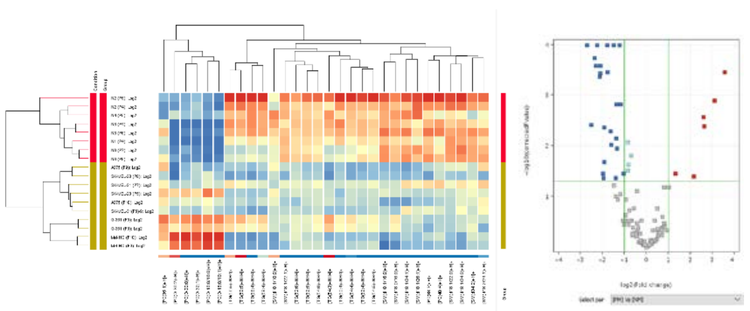	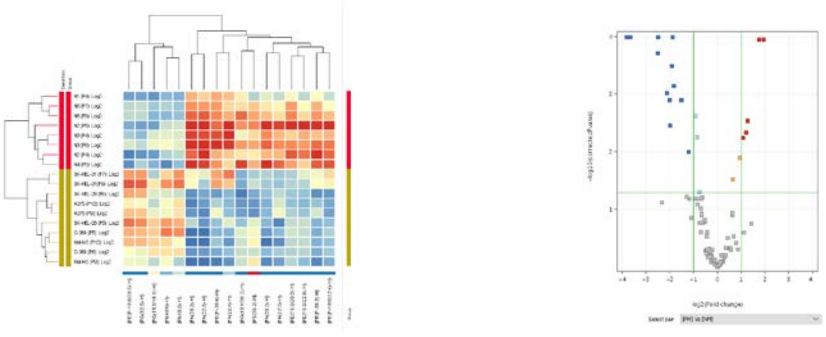
MM vs NM	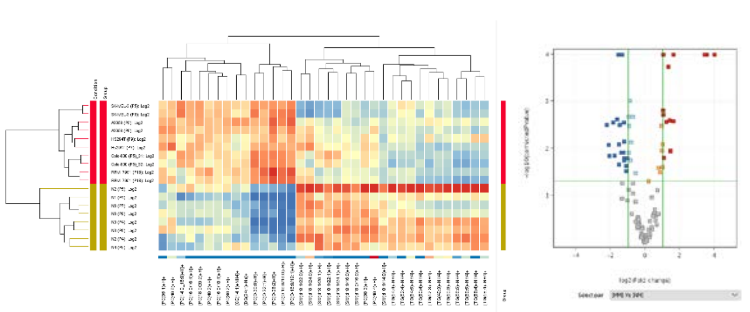	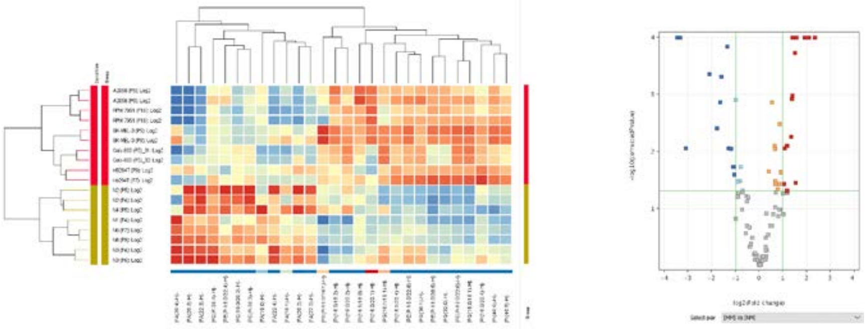
MM vs PM	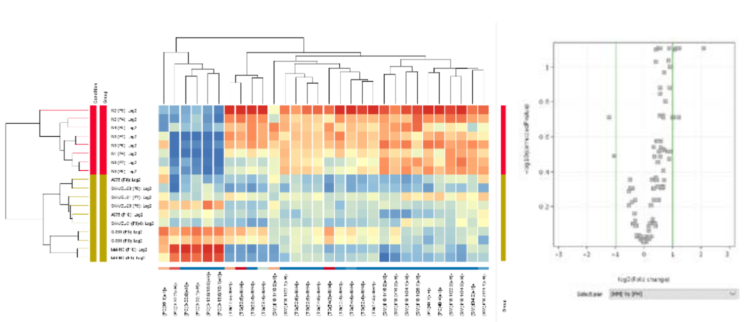	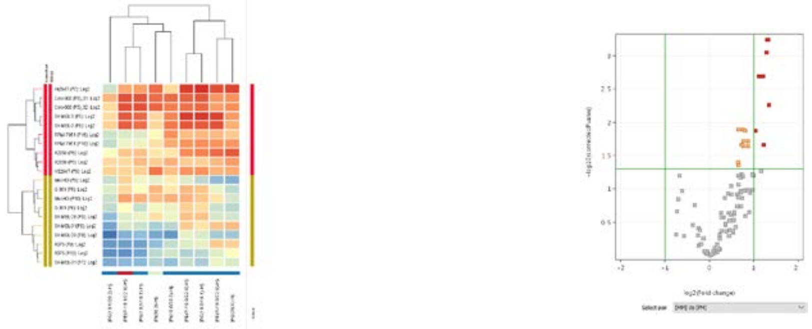
PM+MM vs HeM+NM	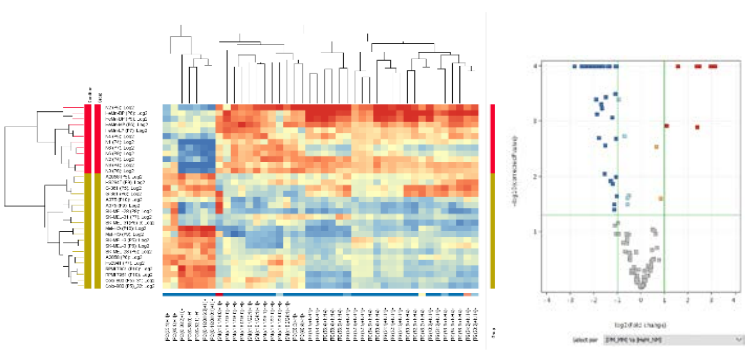	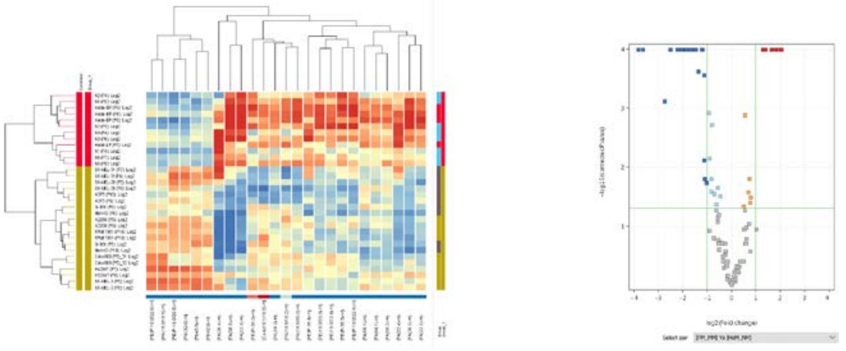

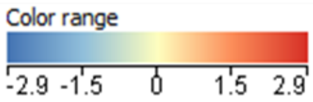

Figure S2

A.

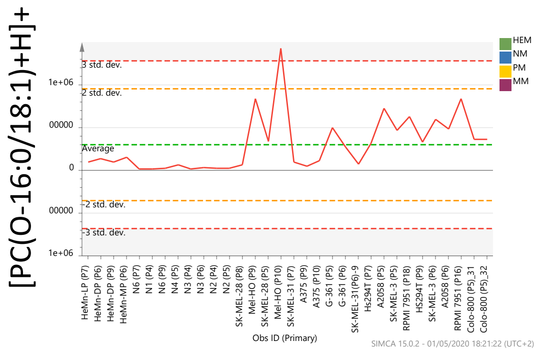

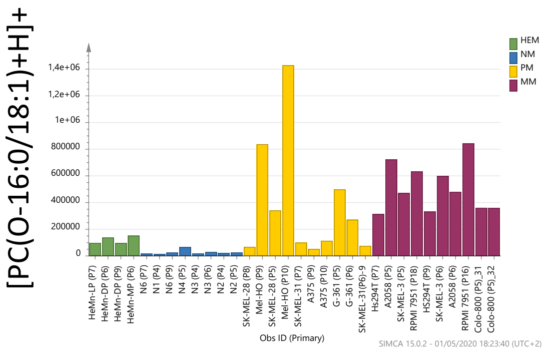

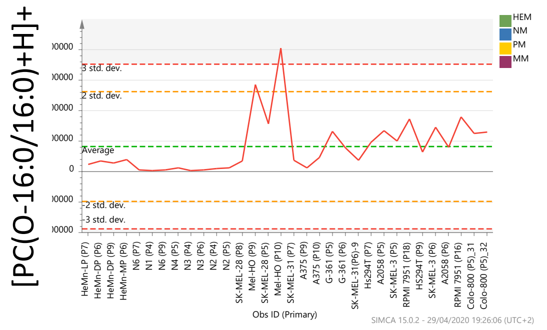

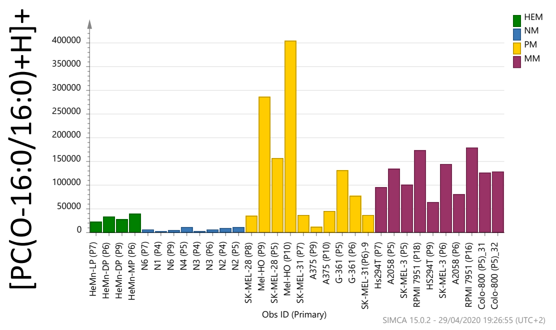

B.

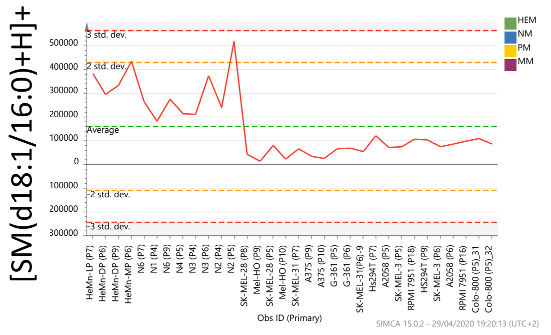

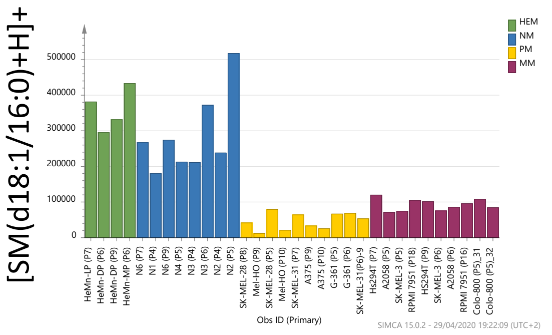

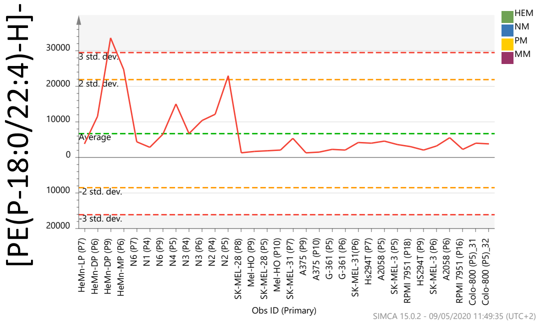

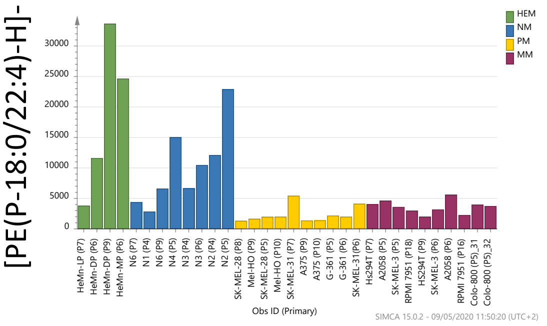

Figure S3

(A)

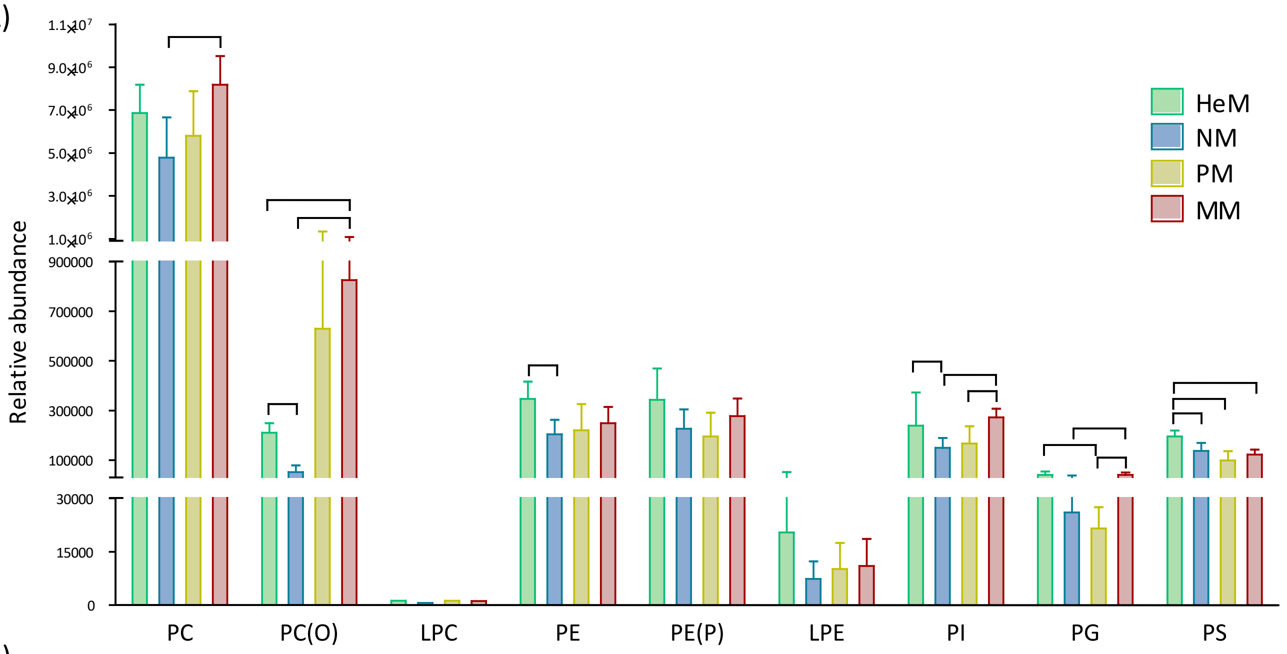

(B)

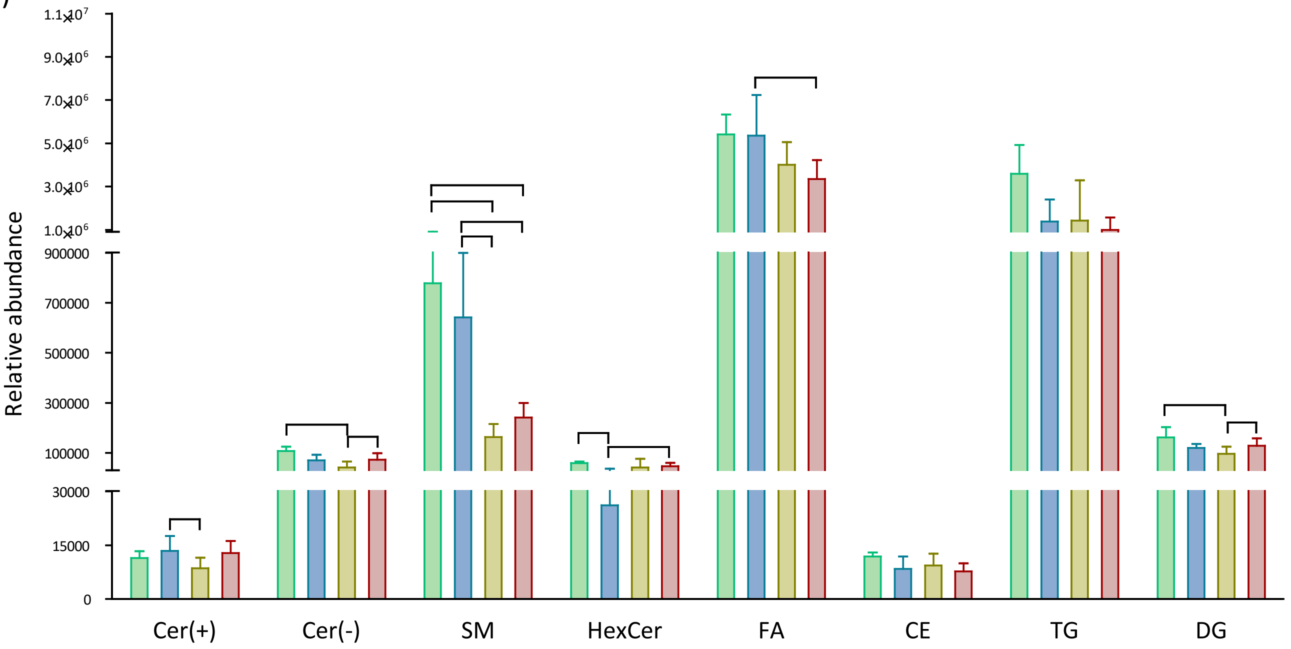

(C)

	Levene Test	One-Way ANOVA	NM vs HeM	PM vs HeM	MM vs HeM	PM vs NM	MM vs NM	MM vs PM	Pair-Wise Minimum Significance
PC	0.058028	0.025037	0.820396	1.000000	1.000000	1.000000	**0.023809**	0.163550	*
ePC[1]	0.002295	0.004154	**0.003823**	0.303206	**0.000452**	0.110637	**0.000103**	0.819341	**
LPC[1]	0.005202	0.173503	0.703362	0.999986	0.999997	0.053760	0.117128	0.999996	NS
PE	0.054649	0.047017	**0.048837**	0.084067	0.300968	1.000000	1.000000	1.000000	*
ePE	0.290922	0.163056	0.706338	0.260618	1.000000	1.000000	1.000000	0.775964	NS
LPE[1]	0.000172	0.385477	0.833663	0.905348	0.930258	0.779711	0.623944	0.987807	NS
PI	0.257654	0.000062	**0.048410**	0.142158	1.000000	1.000000	**0.000169**	**0.000545**	*
PG	0.197118	0.000874	0.191474	**0.027816**	1.000000	1.000000	**0.033217**	**0.001793**	*
PS	0.178572	0.000146	**0.024867**	**0.000074**	**0.002575**	0.086553	1.000000	0.691357	*
Cer[2]	0.499404	0.022457	1.000000	1.000000	1.000000	**0.032903**	1.000000	0.072766	*
Cer[3]	0.960768	0.000656	0.108350	**0.000499**	0.178694	0.107883	1.000000	**0.038422**	*
SM[1]	0.020733	0.000000	0.659795	**0.005799**	**0.007957**	**0.004584**	**0.010377**	0.073908	**
HexCer[1]	0.000100	0.089530	**0.001737**	0.469530	0.194355	0.521126	**0.011556**	0.982406	**
FA	0.076054	0.013212	1.000000	0.398243	0.101830	0.185091	**0.031144**	1.000000	*
CE	0.279629	0.154826	0.372035	0.933733	0.187323	1.000000	1.000000	1.000000	NS
TG[1]	0.049482	0.052177	0.423753	0.463375	0.330199	0.999890	0.841323	0.925995	NS
DG	0.155154	0.002228	0.099773	**0.002171**	0.491368	0.510814	1.000000	**0.045641**	*

Figure S4

A *Fragmentation scheme*

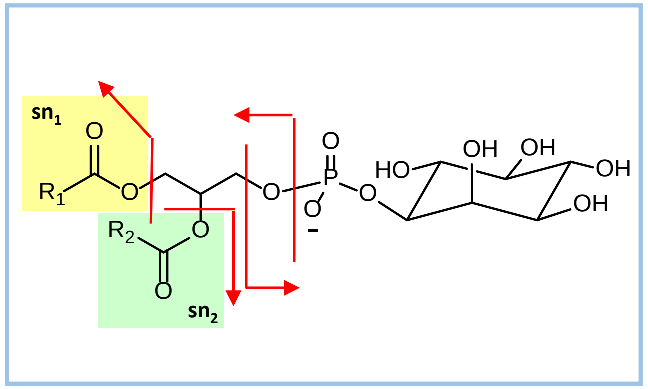

B *Chromatogram*

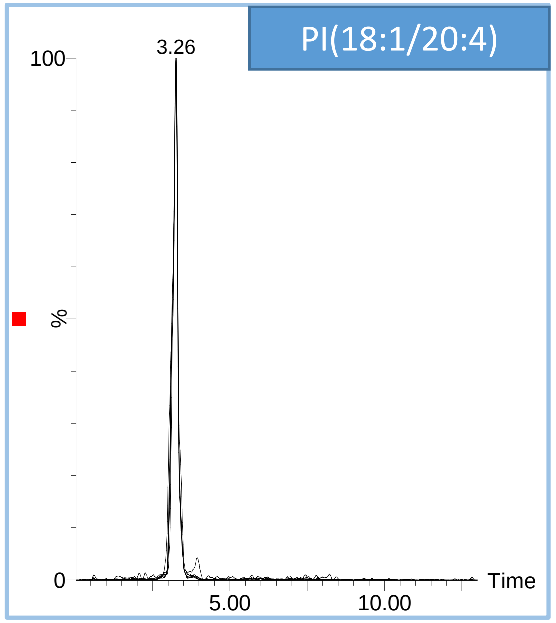

ESI+ MS/MS

C *Spectrum*

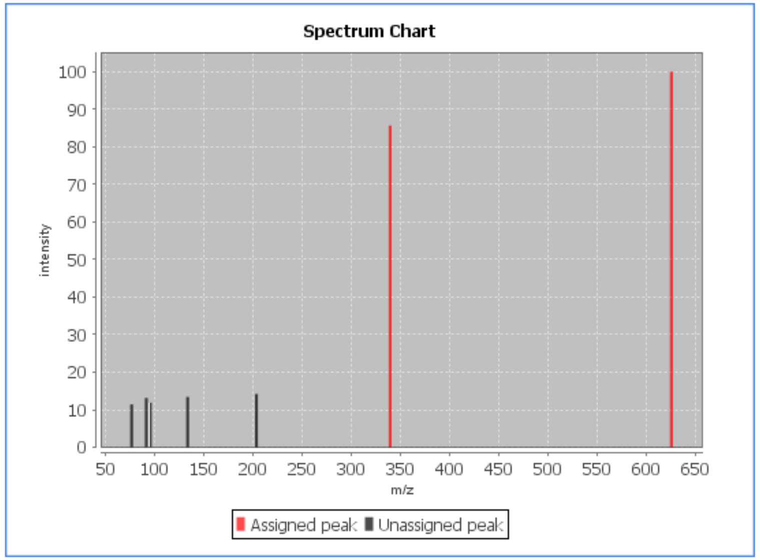

E *Match Lipid*

LipidIon	M-Sc.	T-Sc.	Occ.	St.
PI(18:1_20:4)+NH4	14.9	0.4	74.4	
PI(20:4_18:1)+NH4	14.9	0.4	74.4	
PI(16:0_22:5)+NH4	4	0.4	40.1	
PI(16:1_22:4)+NH4	4	0.4	40.1	
PI(18:0_20:5)+NH4	4	0.4	40.1	
PI(20:3_18:2)+NH4	4	0.4	40.1	
PI(18:3_20:2)+NH4	4	0.4	40.1	
PI(18:4_20:1)+NH4	4	0.4	40.1	
PI(24:1_14:4)+NH4	4	0.4	40.1	
PI(24:2_14:3)+NH4	4	0.4	40.1	
PI(26:1_12:4)+NH4	4	0.4	40.1	
PI(27:1_11:4)+NH4	4	0.4	40.1	
PI(28:1_10:4)+NH4	4	0.4	40.1	

Match Detail

ObsMz	Type	It.(%)	Frag.	Delta(Da)
95.086	MS2	23.005	-	-
131.601	MS2	20.418	-	-
135.1172	MS2	20.065	C10H15	0.0003
220.1337	MS2	41.835	-	-
252.6568	MS2	20.433	-	-
339.2893	MS2	77.027	MG(18:1)-OH	-0.0001
530.7273	MS2	17.41	-	-
625.5189	MS2	100	NL[PI,+NH4]+H	-0.0002

ESI- MS/MS

D *Spectrum*

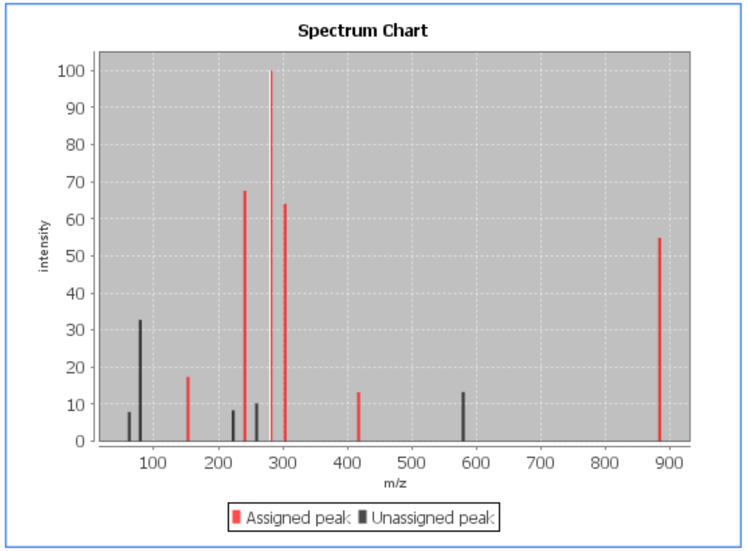

F *Match Lipid*

LipidIon	M-Sc.	T-Sc.	Occ.	St.
PI(18:1_20:4)-H	39.2	0.3	78.3	
PI(20:4_18:1)-H	39.2	0.3	78.3	
PI(16:0_22:5)-H	5.1	0.3	25.4	
PI(16:1_22:4)-H	5.1	0.3	25.4	
PI(18:0_20:5)-H	5.1	0.3	25.4	
PI(20:3_18:2)-H	5.1	0.3	25.4	
PI(18:3_20:2)-H	5.1	0.3	25.4	
PI(18:4_20:1)-H	5.1	0.3	25.4	
PI(24:1_14:4)-H	5.1	0.3	25.4	
PI(24:2_14:3)-H	5.1	0.3	25.4	
PI(26:1_12:4)-H	5.1	0.3	25.4	
PI(27:1_11:4)-H	5.1	0.3	25.4	
PI(28:1_10:4)-H	5.1	0.3	25.4	

Match Detail

ObsMz	Type	It.(%)	Frag.	Delta(Da)
78.9585	MS2	27.457	-	-
152.9949	MS2	29.426	-	-
155.3754	MS2	7.525	-	-
222.9988	MS2	9.456	-	-
241.0127	MS2	66.512	PH(inositol)-H2O-H	0.0008
259.0237	MS2	11.171	IP	0.0012
259.2409	MS2	7.49	-	-
281.2491	MS2	100	FA(18:1)-H	0.0005
303.2329	MS2	66.748	FA(20:4)-H	-0.0001
317.9391	MS2	7.931	-	-
417.2424	MS2	23.367	LPA(18:1)-H3O	0.0012
579.3008	MS2	10.309	-	-
654.6499	MS2	6.671	-	-
883.5335	MS2	77.781	M-H	-0.0007

Figure S5

PC
sn2>sn1

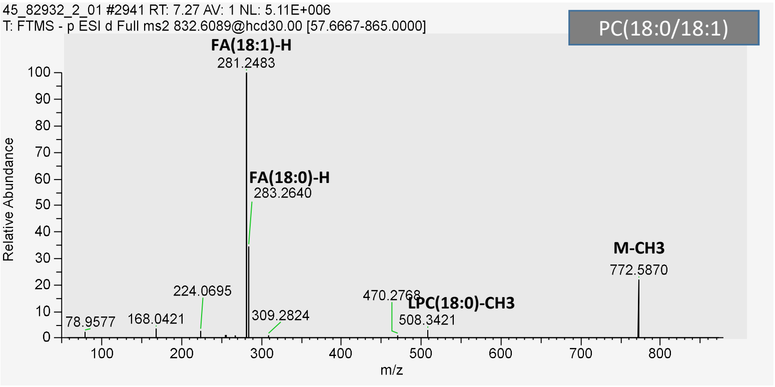

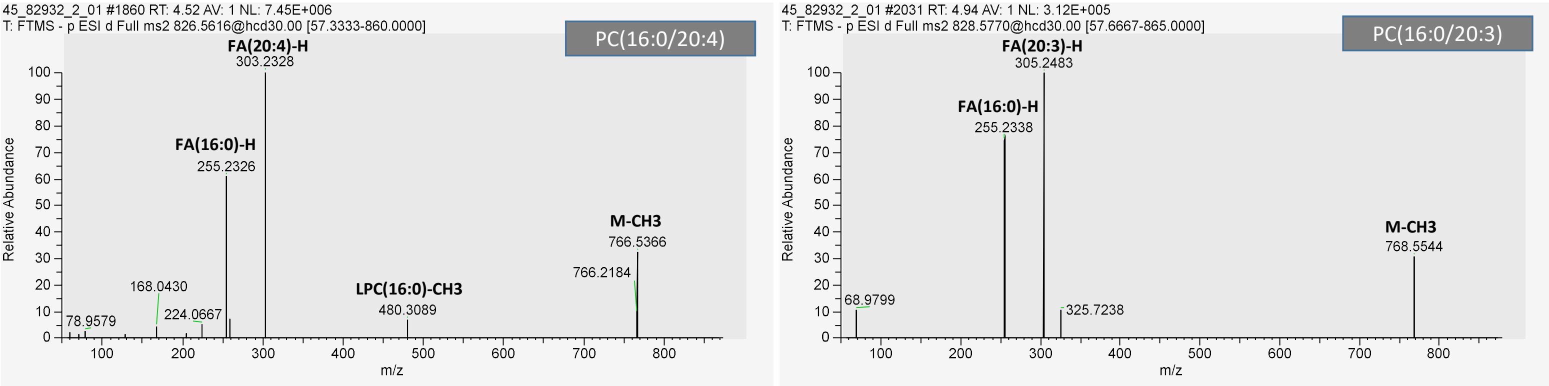

A: MS/MS spectra of the precursor ions of three representative PC species.

PE
sn2>sn1

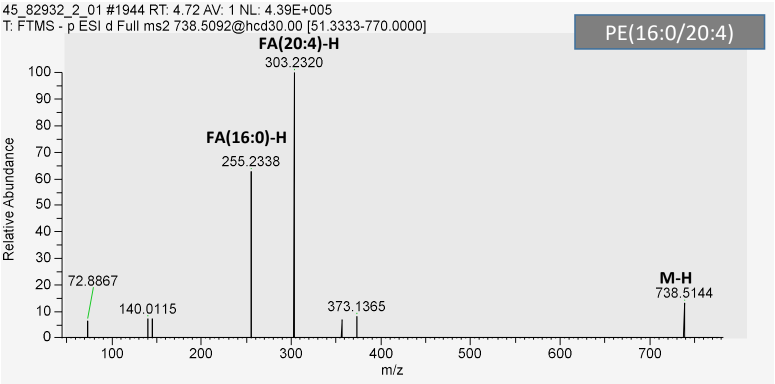

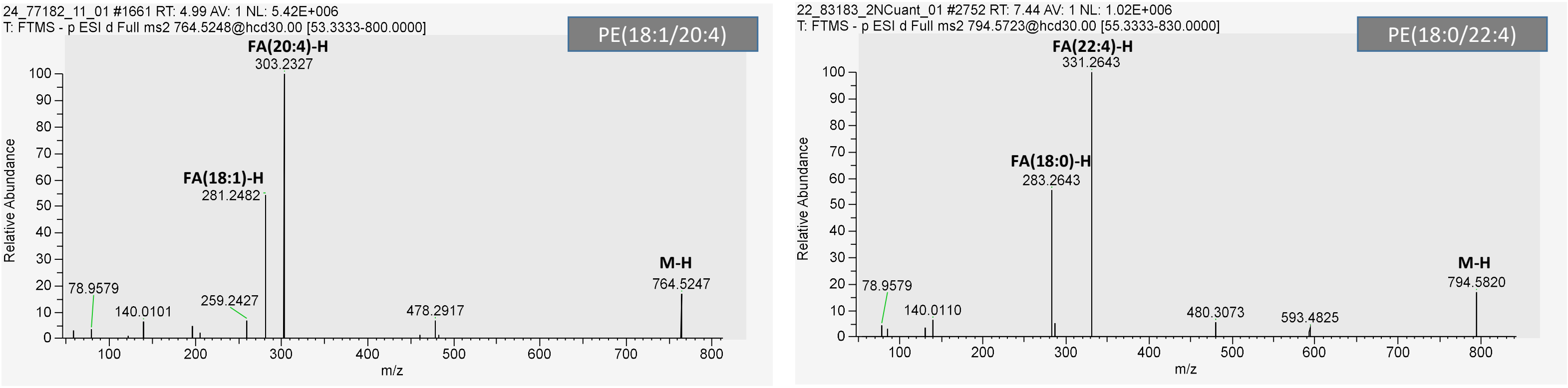

B: MS/MS spectra of the precursor ions of three representative PE species.

PI

sn1>sn2

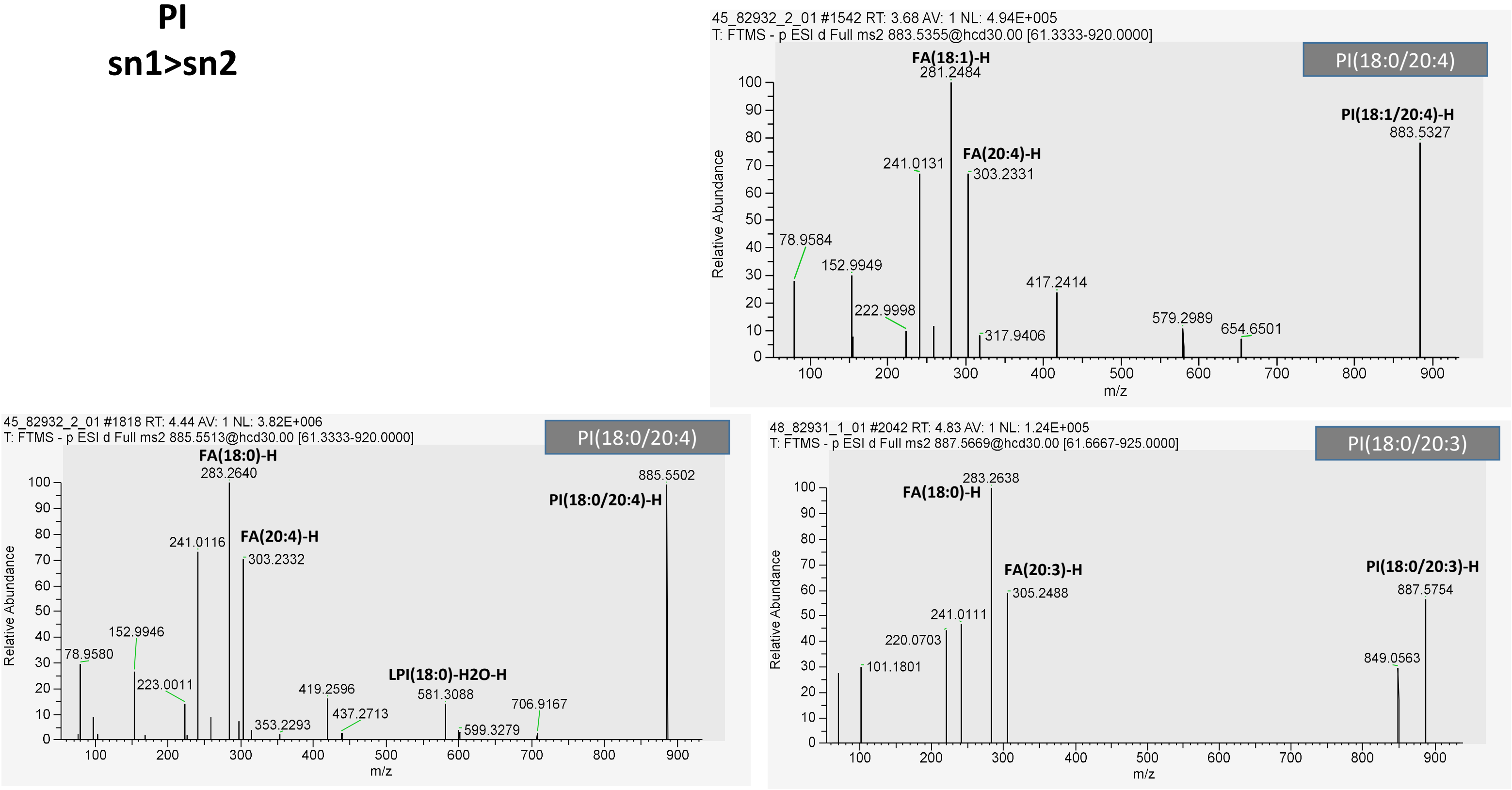

C: MS/MS spectra of the precursor ions of three representative PI species.

PS

sn1>sn2

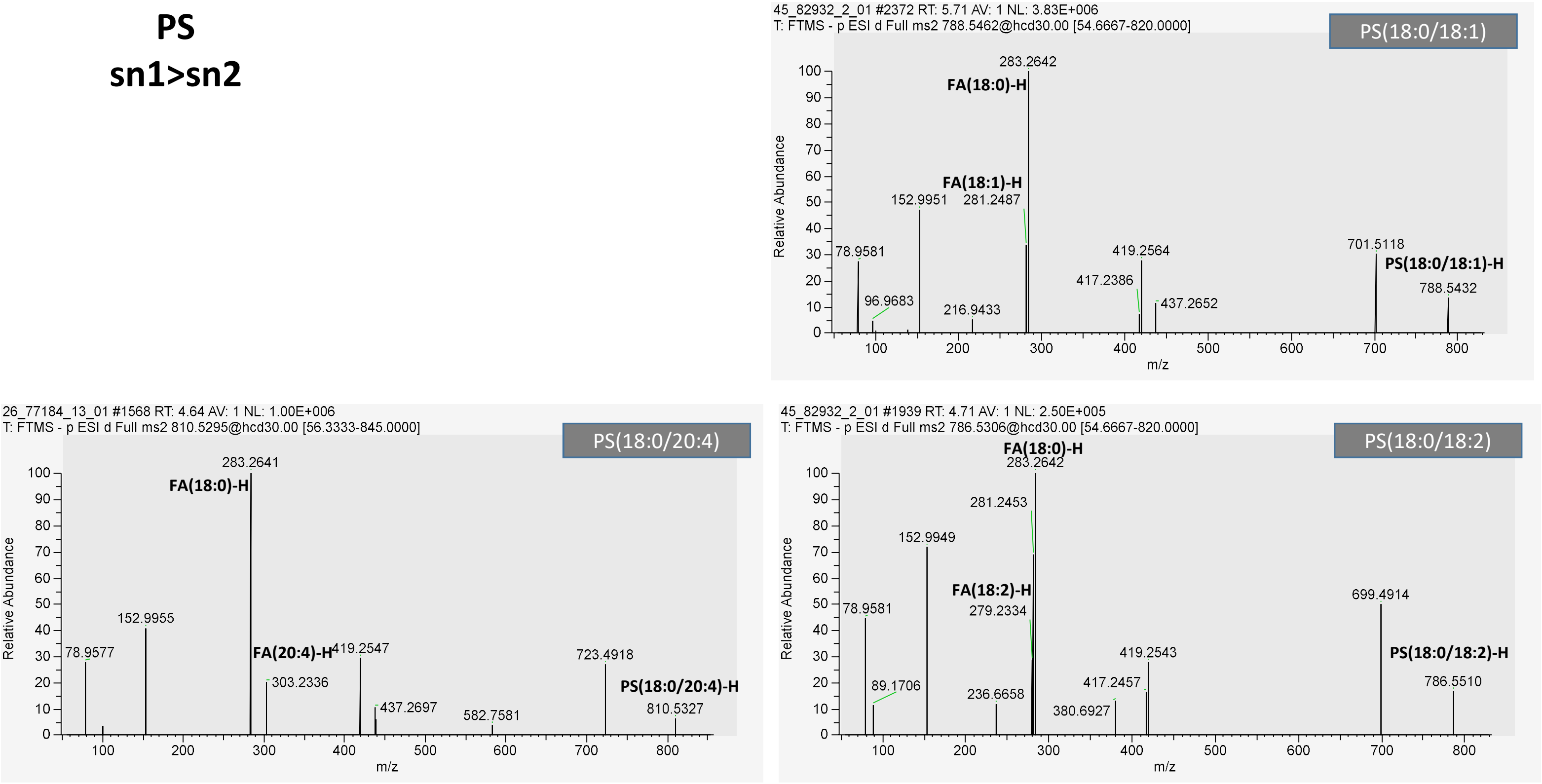

D: MS/MS spectra of the precursor ions of three representative PS species.

PG
sn1>sn2

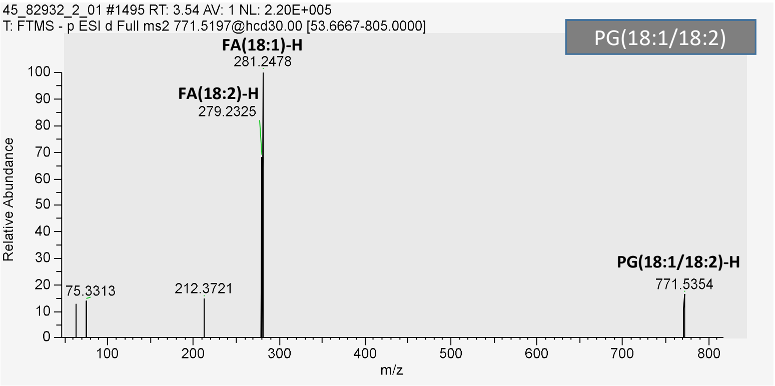

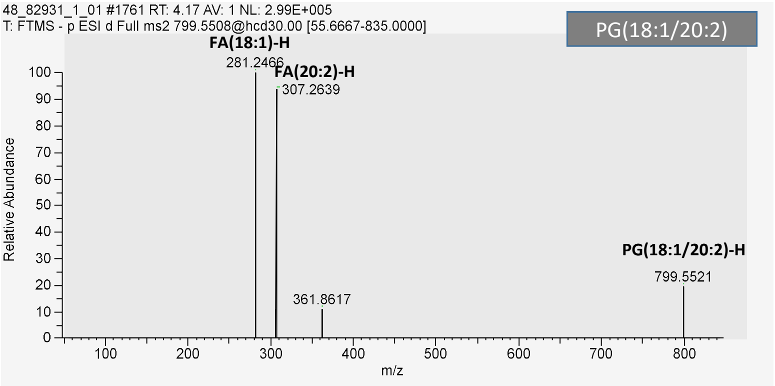

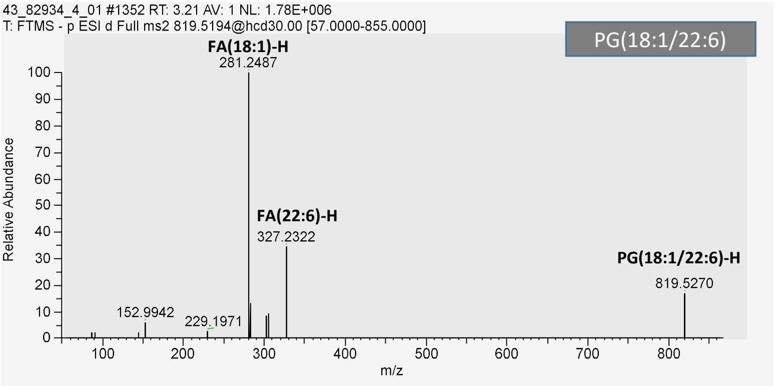

E: MS/MS spectra of the precursor ions of three representative PG species.
